# Causal relationship between educational attainment and the risk of rheumatoid arthritis: a Mendelian randomization study

**DOI:** 10.1186/s41927-021-00216-0

**Published:** 2021-10-21

**Authors:** Guiwu Huang, Jiahao Cai, Wenchang Li, Yanlin Zhong, Weiming Liao, Peihui Wu

**Affiliations:** 1 Department of Joint Surgery, The First Affiliated Hospital of Sun Yat-sen University, Sun Yat-sen University, Guangzhou, China; 2 Department of Neurology, Sun Yat-sen Memorial Hospital, Sun Yat-sen University, Guangzhou, China

**Keywords:** Educational attainment, Rheumatoid arthritis, Mendelian randomization

## Abstract

**Background:**

Educational attainment is moderately heritable and inversely associated with the risk of rheumatoid arthritis. However, the causality from educational attainment on rheumatoid arthritis remained unknown. Here, we aimed to determine whether educational attainment is causally associated with rheumatoid arthritis (RA) by using Mendelian randomization (MR) approach.

**Methods:**

Summary statistics data for RA were obtained from an available, published meta-analysis of genome-wide association studies (GWAS) that included 14,361 RA cases and 43,923 controls of European ancestry. The instrumental variables for educational attainment were obtained from a GWAS meta-analysis that included over 1 million individuals (*N* = 1,131,881) of European ancestry. MR analyses were mainly performed using the inverse-variance weighted (IVW) method. Sensitivity analyses were further performed to test the robustness of the association using the weighted median method, MR-Egger, Cochran Q test, “leave-one-out” analysis and MR-PRESSO test.

**Results:**

A total of 387 SNPs were employed as instrumental variables in our MR analysis. Genetically predicted higher educational attainment was associated with a significantly lower risk of RA using the IVW method (odds ratio [OR] = 0.42, 95% confidence interval [CI]: 0.34–0.52; *p* = 1.78 × 10^− 14^). The weighted median method and MR Egger regression analysis yielded consistent results. The effect estimate remained robust after the outlier variants and SNPs (associated with the confounding factors) were excluded. “Leave-one-out” analysis confirmed the stability of our results. Additionally, the results suggested the absence of the horizontal pleiotropy.

**Conclusions:**

The MR analysis supported a potential inverse causative relationship between educational attainment and the risk of RA.

**Supplementary Information:**

The online version contains supplementary material available at 10.1186/s41927-021-00216-0.

## Background

Rheumatoid arthritis (RA) is a systemic autoimmune disease with a lifetime prevalence of 0.5 ~ 1% worldwide [1, 2]. And it’s characterized by persistent synovitis, progressive joint disability, and extra-articular manifestations [3]. RA results in poor functional status and chronic pain, which erodes the patient’s quality of life, decreases life expectancy and, in some cases, increases mortality [4, 5], leading to extra health expenditures of approximately $19.3 billion per year in the United States [6, 7].

Although the exact cause of RA remains unclear, both environmental and genetic factors contribute to development of the disease. Previous observational epidemiological studies have shown an inverse association between educational attainment and the risk of RA. However, this association may be blurred by the methodological limitations of traditional observational studies, including residual confounding, reverse causation, and measurement error [8].

Educational attainment is a well-established socioeconomic and heritable determinant of health [9], which is defined as years of schooling completed (Edu Years). Educational attainment has been a useful tool in follow-up work to evaluate brain and neural development, biological aging, health behavior and health literacy [10, 11]. However, few studies have assessed the association between educational attainment and RA through genetic variations -- single nucleotide polymorphisms (SNPs).

Mendelian randomization (MR) has been widely used to evaluate causality by exploiting genetic variants with SNPs as instrumental variables to predict the effect of an exposure on a particular outcome [12], which overcomes the typical pitfalls such as reverse causation and confounders that hinder observational studies [13, 14]. Herein, we performed the MR approach to analyze the potential causal effect of educational attainment on the risk of RA.

## Methods

### Study overview

**Fig. 1:**
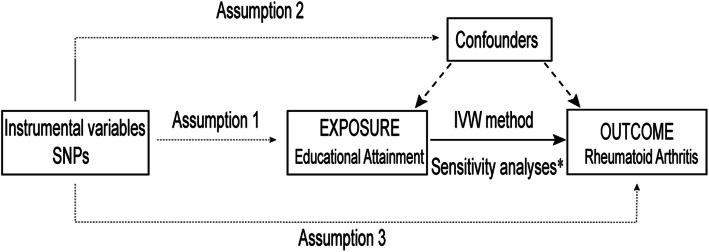
An overview of study design. SNP, single nucleotide polymorphism; IVW, inverse-variance weighted

This study applied MR as a method to determine whether educational attainment is causally associated with RA, using summary data of SNP-exposure (educational attainment) and SNP-outcome (rheumatoid arthritis) based on genome-wide association studies (GWAS). An overview of the study design is shown in Fig. 1.

Assumption 1, genetic variants should be strongly associated with the exposure; Assumption 2, genetic variants extracted for exposure should be independent of any confounder which is associated with both exposure and outcome; and Assumption 3, the genetic variants affect the outcome only through the exposure. *Sensitivity analyses: Weight median method, MR-Egger regression, MR-PRESSO, Leave-one-out test.

### RA GWAS summary statistics

Publicly available summary statistic estimates for the associations between genetic variants and risk of RA were obtained from a GWAS meta-analysis, including 58,284 individuals from 18 studies of European ancestry (14,361 RA cases and 43,923 controls) [15]. All RA cases fulfilled the 1987 RA diagnosis criteria of the American College of Rheumatology [16] or were diagnosed as RA by a professional rheumatologist. Among all RA cases enrolled in this study, 88.1% were seropositive and 9.3% were seronegative for anti-citrullinated peptide antibody (ACPA) or rheumatoid factor (RF), and 2.6% had unknown autoantibody status.

### Selection of instrumental variables

The genetic instrumental variables associated with educational attainment were obtained from a GWAS meta-analysis [17] comprising 71 quality-controlled, cohort-level results files, with a sample size of more than 1 million individuals of European ancestry (*N* = 1,131,881). All cohort-level analyses were restricted to European-ancestry individuals who passed the cohort’s quality control and whose educational years were measured at an age of at least 30. The phenotype was constructed by mapping each major educational qualification that can be identified from the cohort’s survey measure to an International Standard Classification of Education (ISCED) category and imputing a years-of-education equivalent for each ISCED category. In this study, the educational attainment was evaluated by years of schooling completed (Edu Years, mean ± standard deviation (SD) = 16.8 ± 4.2 years).

A number of quality control steps were taken in our analysis to select eligible instrumental SNPs that were strongly associated with educational attainment. First, we identified 1271 lead SNPs at the genome-wide significance threshold (*p* < 5 × 10^− 8^). After clumping correlated SNPs (linkage disequilibrium [LD] r^2^ ≥ 0.001), 393 SNPs remained and were used as instrumental variables. We then extracted educational attainment–associated SNPs from the outcome data (RA, in this study). For SNPs absent in the outcome data, we identified proxy SNPs at a cutoff of LD of r^2^ > 0.8 from the SNiPA website (https://snipa.helmholtz-muenchen.de/snipa3/index.php). SNPs missing in the outcome data without appropriate proxy SNPs available were then excluded. We then calculated the *F* statistic for each of the SNPs using the following formula: *R*^2^ × (*N* − 2)/(1 − *R*^2^) . Here, R^2^ indicates the proportion of variance in educational attainment explained by a given SNP and N indicates sample size. More specifically, R^2^ was calculated with the following formula: *R*^2^ = [2 × Beta^2^ × (1 − EAF) × EAF]/[2 × Beta^2^ × (1 − EAF) × EAF + 2 × SE^2^ × *N* × (1 − EAF) × EAF]. Here, Beta indicates the genetic effect of SNP on educational attainment, EAF is effect allele frequency, SE is standard error and N is sample size. *F* statistic is recommended to be over 10 to avoid employing week genetic instruments [18].

### Statistical analyses

Statistical analyses were performed using the two-sample MR package (version 0.5.5) in R software version 4.0.2 (https://www.r-project.org/); *p* < 0.05 was the threshold for a significant difference. All estimates were reported with two-tailed *p*-values. In the main analysis, we utilized the inverse-variance weighted (IVW) method to investigate the causality between educational attainment and RA [19–21]. And the results were expressed as OR per one SD change in years of education. And one SD in years of education was 4.2 years.

To evaluate potential pleiotropy, weighted median and MR-Egger methods were used as sensitivity analyses. And we detected directional pleiotropy using intercept derived from MR-Egger regression [20]. Then we evaluated the heterogeneity using Cochran Q test. We also performed leave-one-out analysis to evaluate whether the observed causal relationship was reliant on any single SNP. Finally, MR-PRESSO test [22] was conducted to detect any outlier with potential pleiotropy. Once the outliers were identified, we removed them and repeated MR analysis.

Then we retrieved previously published MR studies related to RA from PubMed and identified the potential risk factors causally associated with RA (Vitamin D, body mass index, smoking, alcohol consumption, coffee consumption, mineral nutrients, gut microbiome, diet). These risk factors might be potential confounding factors of this MR study. And confounders considered in this study should be associated with both RA and educational attainment. Therefore, we further conducted a comprehensive search in the GWAS Catalog (http://www.ebi.ac.uk/gwas; accessed on November 17, 2020) for whether any SNP in this study was associated with these confounders at the genome-wide significance of *p* < 5 × 10^− 8^. Then we found 18 SNPs associated with confounders (smoking initiation [23], body mass index (BMI) [24], and type 2 diabetes mellitus [25], shown in Additional file 1: table S4). As for the other SNPs, we did not find these SNPs were associated with confounding factors. Therefore, we only selected smoking, BMI, and diabetes as confounders in our study. Analyses were performed again to test whether the association remained significant after excluding 18 SNPs associated with RA and risk factors other than educational attainment.

## Results

A total of 1271 lead SNPs were identified at the genome-wide significance threshold (*p* < 5 × 10^− 8^) and 393 SNPs remained after clumping (r^2^ < 0.001). However, seven SNPs were not available in the summary statistics data for RA. Of these, one proxy variant (rs11212135, LD r^2^ = 0.82) was identified for the missing SNP (rs72486027). Six SNPs (rs11657342, rs182902112, rs73581580, rs75033012, rs75177132, rs76246107) without appropriate proxy SNPs available were excluded. And after the harmonizing process, one SNP (rs77719387) was removed for incompatible alleles and 13 SNPs were excluded for being palindromic with intermediate allele frequencies (Additional file, Table 1). Therefore, 373 SNPs were chosen as instrumental variables for educational attainment in the present study (Additional file, Table 2). The SNP rs2256965 was the only outlier variant detected by the MR-PRESSO test.

**Fig. 2:**
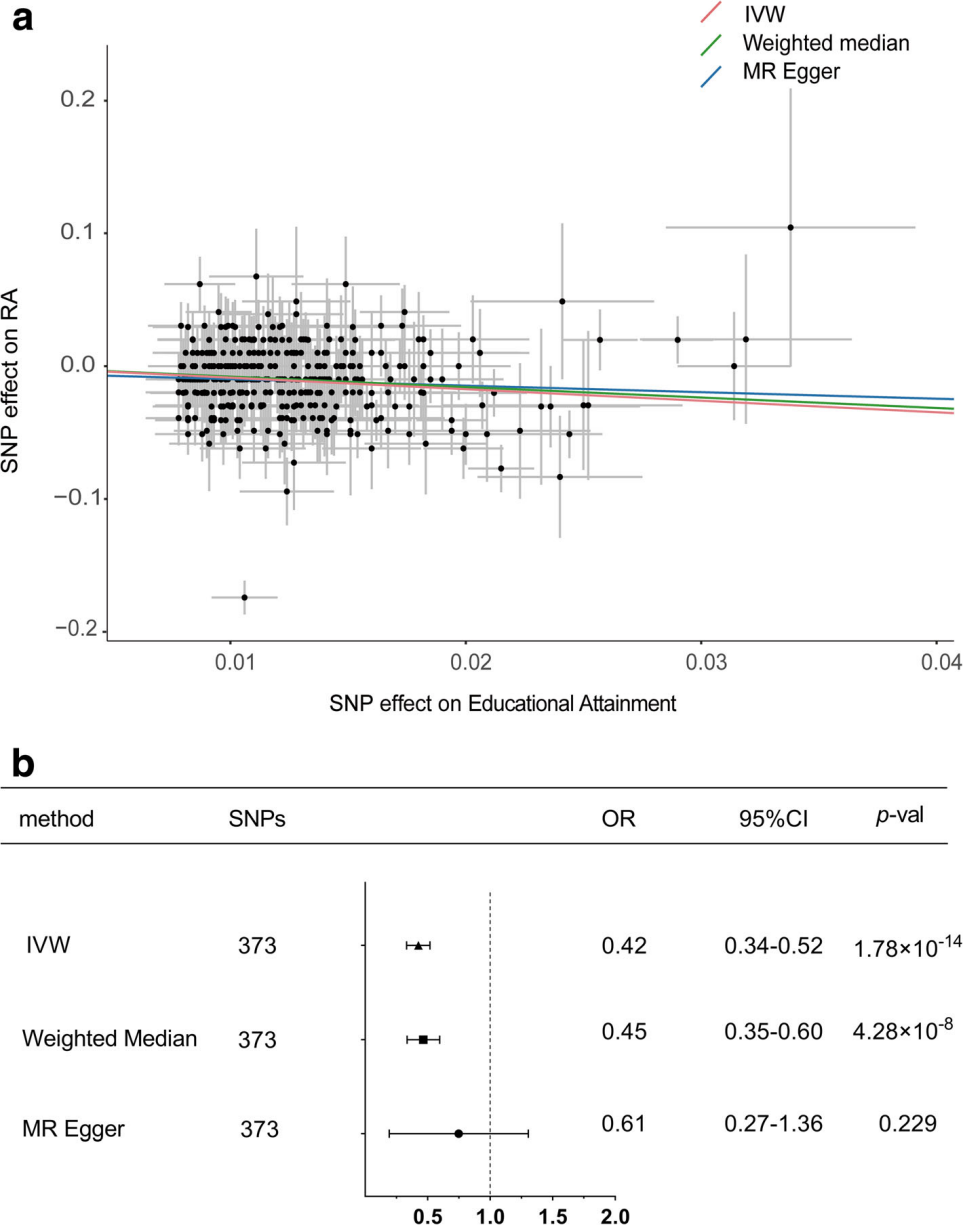
**A** Scatter plot and (**B**) Forest plot of Mendelian randomization analyses for the associations of educational attainment with risk of rheumatoid arthritis. OR, odds ratio; CI, confidence interval; IVW, inverse-variance weighted method; MR, Mendelian randomization; SNP, single nucleotide polymorphism; *p*-val, *p* value

As were shown in Fig. 2, along with the 373 stringently selected SNPs for subsequent two-sample MR analysis, we found strong evidence to support a causal association between educational attainment and RA using the IVW method (odds ratio [OR] = 0.42, 95% confidence interval [CI]: 0.34–0.52; *p* = 1.78 × 10^− 14^), which meant that RA risk decreased by 58% per SD (approximately 4.2 years) increased in the years of schooling. The associations were consistent in the sensitivity analysis using the weighted median method (OR = 0.45, 95% CI: 0.34–0.60; *p* = 4.27 × 10^− 8^). The effect was only slightly attenuated using the MR-Egger method (OR = 0.61; 95% CI: 0.27–1.36; *p* = 0.229). Considering that the weighted median estimator had the advantage of retaining greater precision of the estimates compared with the MR-Egger analysis [19] and MR-Egger method was often used as a reference for the direction of causal association the results of the MR analysis, we believe that our study supports an inversely causative relationship between educational attainment and RA. Furthermore, the association estimated by the IVW method was markedly significant after correction for one outlier variant detected by MR-PRESSO test (OR = 0.46, 95% CI: 0.38–0.55, *p* = 4.89 × 10^− 16^).

Heterogeneity tests suggested an apparent sign of heterogeneity: Q value (df) = 594.21(372), *p* = 1.82 × 10^− 12^. However, after removing the outlying SNP, heterogeneity was remarkably decreased: Q value (df) = 425.7(371), *p* = 0.03. Additionally, we found no indication of unbalanced pleiotropy (*p*-value for Egger intercept = 0.34). The results of the leave-one-out analysis (Additional file, Table 3) demonstrated no potentially influential SNPs driving the causal link between educational attainment and RA in the replicated analyses.

We then scanned the SNPs for their potential secondary phenotypes using the GWAS catalog. As was shown in Additional file, Table 4, a total of 18 SNPs associated with educational attainment were found to be associated with other traits affecting RA. After excluding SNPs associated with potential confounders, results from the statistical analysis remained essentially consistent (OR = 0.45, 95% CI: 0.37–0.55, *p* = 1.05 × 10^− 15^, using the IVW method).

## Discussion

In this two-sample MR analysis, we leveraged the largest genetic data set for educational attainment published to date, together with the largest GWAS addressing the outcome of interest, to understand causal relationships between educational attainment and risk of RA. We identified a pronounced causal effect that genetic predisposition to higher educational attainment was associated with a lower risk of RA.

In fact, education inequalities in risk of RA have long been noted. Pincus and colleagues [26] identified an association between a lower level of formal education and higher mortality and morbidity related to RA over a 9-year period. Another study found that formal education level can be a significant marker of clinical status in RA [27]. However, some studies have revealed that level of formal education is not significantly associated with risk of RA [28, 29].

Given that these studies with inconsistent conclusions were either based on limited samples or only explored correlations from epidemiological observational studies, few studies have clearly and consistently demonstrated a biological link underlying this association. By applying MR analysis in the current study to alleviate these problems, we provided concrete evidence to support an inverse causal association of educational attainment with risk of RA. The credibility of this study was verified by using several data sets with largest sample size.

A previous two-sample MR study conducted by Bae and Lee [30] suggested that RA risk decreased by 52% per SD (approximately 3.61 years) increased in the years of schooling completed used relatively small sample size statistical data set of years of education from the UK Biobank GWAS (*n* = 293,723) as the exposure and a meta-analysis of GWAS of RA (*n* = 5539) and European controls (*n* = 20,169) as the outcome. Here, we expanded the exposure sample size to over 1 million individuals (*N* = 1,131,881) and chose the latest meta-analysis of GWAS, which included 58,284 individuals of European ancestry (14,361 RA cases and 43,923 controls). Meanwhile, the number of SNPs chosen as instrumental variables increased dramatically (from 49 to 373). Furthermore, individuals with RA who were seropositive and seronegative for ACPA or RF were enrolled in this MR analysis. Recently, a comparable analysis has been carried out by Yuan et al. used the same GWAS as sources for educational attainment (EA, exposure) and rheumatic arthritis (RA, outcome) [31]. And they found that RA risk decreased by 50% per SD (approximately 4.2 years) increased in the years of schooling completed. However, there are some differences that should be noticeable between our study and the Yuan’s. Firstly, the linkage disequilibrium (LD) r^2^ of the SNPs in our study was set to a more conservative threshold to obtain a smaller set of SNPs (LD r^2^ < 0.001 with 387 SNPs remained vs. LD r^2^ < 0.01 with 663 SNPs used in Yuan’s study) to ensure the independence of SNPs at the cost of decreasing statistical power. Secondly, the RA phenotype used in Yuan’s study came from mixed populations (European and Asian ancestry). However, it should be noticed that population heterogeneity may lead to bias to the MR results. As such, all the association analyses performed in our study were restricted to European-descent individuals, making the MR estimates more reliable. As such, our MR study is based on merely European descents and a set of more conservative genetic instruments, to appraise the causal relationship between EA and the risk of RA. Thus, the causative association fully explored in patients between educational attainment and risk of RA was more convincing.

In total, the identified exposure SNPs accounted for approximately 11% of the variance in educational attainment. The effect size of the independent SNPs corresponding to an educational increase was obtained as follows: the median effect size corresponded to 1.7 weeks of schooling per allele (95% CI: 1.1–2.6 weeks). Furthermore, the genes related to these SNPs are involved in almost all aspects of neuron-to-neuron communication [17]. The dramatic increase in our sample size enabled us to improve the power of the test.

The MR approach, as an approximation to a randomized controlled trial in nature, offers one of the most compelling methods to detect causation. The IVW method and weighted median method suggested an inverse causal association between educational attainment and RA, whereas the MR-Egger method showed no proof of a causative association between educational attainment and RA. However, the MR-Egger test provided a reference for the direction of causal association. The weighted median method, which is not influenced by outlying genetic variants, improved the power of causal effect detection and effectively decreased type I error [19]. Therefore, the weighted median method had a distinct advantage over the MR-Egger test, and its result in this study was the same as that of the IVW method.

The results of our MR analysis might be biased by pleiotropy. Heterogeneity tests suggested an apparent sign of heterogeneity (Q value (df) = 594.21(372), *p* = 1.82 × 10^− 12^). However, heterogeneity was decreased after removing the outlying SNP detected by MR PRESSO test (Q value (df) = 425.7(371), *p* = 0.03). Additionally, there was no indication of unbalanced pleiotropy (*p*-value for MR Egger intercept = 0.34). Therefore, we deemed that the conclusion would not be biased significantly by the heterogeneity of the analysis because several robust methods were performed, which could provide reliable inferences and statistical support.

The potential mechanisms that educational attainment ultimately reduces the risk of RA may be complicated. In general, higher educational attainment is associated with greater wealth and status, as well as healthier lifestyle and relatively higher quality of life. This cascade of benefits from higher educational attainment may eventually contribute to RA prevention. In addition, the effect of educational attainment on RA may also be mediated by obesity. In an MR study, Böckerman et al. found that the higher years of schooling was associated with lower BMI [32]. Another MR study has also shown that higher years of schooling was associated with lower plasma triglyceride levels, waist circumference and waist-to-hip ratio [33]. This study suggests that higher education attainment can protect against obesity to some extent and higher educational attainment could be a protective factor against obesity in advanced countries [32]. What’s more, previous study has shown that lower educational attainment was associated with an increased risk of Type 2 Diabetes Mellitus (T2DM) [33]. And T2DM was a significant risk factor for RA [25, 34]. And Qian et al. has found that genetic predisposition to smoking was positively associated with rheumatoid arthritis [23]. To some extent, some studies have found that well-educated individuals were less likely to smoke [35–37]. Based on the points discussed above, the influence of educational attainment on individuals has multiple aspects, and the total vector effects reflected in RA is the preventive effect. However, the potential biological mechanisms were rarely reported. Further researches are needed to uncover the pathways about how the educational attainment decreases the risk of RA.

This study has several limitations. The summary GWAS data were restricted to individuals of European descent, and, because ethnicity may affect causality, our results may not be fully representative of the non-European populations. Another limiting factor was that this applied analysis could not be stratified by gender and age due to the meta-GWAS was performed without adjustment for gender or age to maximize statistical power, thus, we could not assess gender or age discrepancies and potential nonlinear associations.

In conclusion, our aim in this study was to assess the causal effect of educational attainment and risk of RA by using two-sample MR analysis with pretty large sample sizes. However, further confirmatory methods should be conducted to verify our findings of a potential causal association between increased educational attainment and lower risk of RA. These results advocate the current clinical practice for RA surveillance in those with lower educational attainment.

## Supplementary Information

**Additional file 1: Table S1.** Detailed information of SNPs Harmonizing. **Table 2.** Detailed information of LD-independent SNPs chosen as instrumental variables for educational attainment (exp) and rheumatoid arthritis (out). **Table 3.** Detailed information of the “leave-one-out” analysis corresponding to the IVW analysis. **Table 4.** The SNPs and their corresponding phenotypes considered as confounders.

## Data Availability

The datasets generated and/or analysed during the current study are publicly available and included in this published article and its supplementary information files.

## Abbreviations

MR: Mendelian randomization
GWAS: Genome-wide association studies
SNPs: Single nucleotide polymorphisms
RA: Rheumatoid arthritis
IVW: Inverse-variance weighted
CI: Confidence interval

## References

[1] Aletaha D, Smolen JS (2018). Diagnosis and Management of Rheumatoid Arthritis: a review. JAMA.

[2] Smolen JS, Aletaha D, McInnes IB (2016). Rheumatoid arthritis. Lancet.

[3] Scott D, Wolfe F, Huizinga T (2010). Rheumatoid arthritis. Lancet.

[4] England B, Sayles H, Michaud K (2016). Cause-specific mortality in male US veterans with rheumatoid arthritis. Arthritis Care Res.

[5] Verstappen S (2015). Rheumatoid arthritis and work: the impact of rheumatoid arthritis on absenteeism and presenteeism. Best Pract Res Clin Rheumatol.

[6] Birnbaum H, Pike C, Kaufman R, Maynchenko M, Kidolezi Y, Cifaldi M (2010). Societal cost of rheumatoid arthritis patients in the US. Curr Med Res Opin.

[7] Cross M, Smith E, Hoy D, Carmona L, Wolfe F, Vos T, Williams B, Gabriel S, Lassere M, Johns N, Buchbinder R, Woolf A, March L (2014). The global burden of rheumatoid arthritis: estimates from the global burden of disease 2010 study. Ann Rheum Dis.

[8] Boyko E (2013). Observational research--opportunities and limitations. J Diabetes Complicat.

[9] Heath A, Berg K, Eaves L (1985). Education policy and the heritability of educational attainment. Nature.

[10] Anttila V, Bulik-Sullivan B, Finucane H (2018). Analysis of shared heritability in common disorders of the brain. Science.

[11] Marioni R, Ritchie S, Joshi P (2016). Genetic variants linked to education predict longevity. Proc Natl Acad Sci U S A.

[12] Davies N, Holmes M, Davey SG (2018). Reading Mendelian randomisation studies: a guide, glossary, and checklist for clinicians. BMJ.

[13] Sekula P, Del Greco MF, Pattaro C (2016). Mendelian randomization as an approach to assess causality using observational data. J Am Soc Nephrol.

[14] Emdin C, Khera A, Kathiresan S (2017). Mendelian randomization. JAMA.

[15] Okada Y, Wu D, Trynka G (2014). Genetics of rheumatoid arthritis contributes to biology and drug discovery. Nature.

[16] Arnett F, Edworthy S, Bloch D (1988). The American rheumatism association 1987 revised criteria for the classification of rheumatoid arthritis. Arthritis Rheum.

[17] Lee J, Wedow R, Okbay A (2018). Gene discovery and polygenic prediction from a genome-wide association study of educational attainment in 1.1 million individuals. Nat Genet.

[18] Pierce B, Burgess S (2013). Efficient design for Mendelian randomization studies: subsample and 2-sample instrumental variable estimators. Am J Epidemiol.

[19] Bowden J, Davey Smith G, Haycock P (2016). Consistent estimation in Mendelian randomization with some invalid instruments using a weighted median estimator. Genet Epidemiol.

[20] Burgess S, Thompson S (2017). Interpreting findings from Mendelian randomization using the MR-egger method. Eur J Epidemiol.

[21] Hemani G, Zheng J, Elsworth B, Wade KH, Haberland V, Baird D, et al. The MR-base platform supports systematic causal inference across the human phenome. eLife. 2018;7. 10.7554/eLife.34408. 10.7554/eLife.34408 PMC5976434 29846171

[22] Verbanck M, Chen CY, Neale B, Do R (2018). Detection of widespread horizontal pleiotropy in causal relationships inferred from Mendelian randomization between complex traits and diseases. Nat Genet.

[23] Qian Y, Zhang L, Wu D (2020). Genetic predisposition to smoking is associated with risk of rheumatoid arthritis: a Mendelian randomization study. Arthritis Res Ther.

[24] Bae S, Lee Y (2019). Causal association between body mass index and risk of rheumatoid arthritis: a Mendelian randomization study. Eur J Clin Investig.

[25] Inamo J, Kochi Y, Takeuchi T (2020). Is type 2 diabetes mellitus an inverse risk factor for the development of rheumatoid arthritis?. J Hum Genet.

[26] Pincus T, Callahan L, Burkhauser R (1987). Most chronic diseases are reported more frequently by individuals with fewer than 12 years of formal education in the age 18-64 United States population. J Chronic Dis.

[27] Pincus T, Callahan L (1985). Formal education as a marker for increased mortality and morbidity in rheumatoid arthritis. J Chronic Dis.

[28] Uhlig T, Hagen K, Kvien T (1999). Current tobacco smoking, formal education, and the risk of rheumatoid arthritis [J]. J Rheumatol.

[29] Bankhead C, Silman A, Barrett B, Scott D, Symmons D (1996). Incidence of rheumatoid arthritis is not related to indicators of socioeconomic deprivation. J Rheumatol.

[30] Bae S, Lee Y (2019). Causal relationship between years of education and the occurrence of rheumatoid arthritis. Postgrad Med J.

[31] Yuan S, Xiong Y, Michaëlsson M, Michaëlsson K, Larsson SC (2021). Genetically predicted education attainment in relation to somatic and mental health. Sci Rep.

[32] Böckerman P, Viinikainen J, Pulkki-Råback L, Hakulinen C, Pitkänen N, Lehtimäki T, Pehkonen J, Raitakari OT (2017). Does higher education protect against obesity? Evidence using Mendelian randomization. Prev Med.

[33] Liao LZ, Chen ZC, Li WD, Zhuang XD, Liao XX (2021). Causal effect of education on type 2 diabetes: a network Mendelian randomization study. World J Diabetes.

[34] Jiang P, Li H, Li X (2015). Diabetes mellitus risk factors in rheumatoid arthritis: a systematic review and meta-analysis. Clin Exp Rheumatol.

[35] Kim JH, Noh J, Choi JW (2017). Association of education and smoking status on risk of diabetes mellitus: a population-based nationwide cross-sectional study. Int J Environ Res Public Health.

[36] de Walque D (2007). Does education affect smoking behaviors? Evidence using the Vietnam draft as an instrument for college education. J Health Econ.

[37] Lawrence EM (2017). Why do College graduates behave more healthfully than those who are less educated?. J Health Soc Behav.

