# Supplementary material for: Causal relationship between educational attainment and the risk of rheumatoid arthritis: a Mendelian randomization study
Source: BMC Rheumatol. 2021 Oct 21;5:47. doi: 10.1186/s41927-021-00216-0 (PMC8529827; doi:10.1186/s41927-021-00216-0)
Supplement: Supplementary file 1 — Additional file 1: Table S1. Detailed information of SNPs Harmonizing. Table 2. Detailed information of LD-independent SNPs chosen as instrumental variables for educational attainment (exp) and rheumatoid arthritis (out). Table 3. Detailed information of the “leave-one-out” analysis corresponding to the IVW analysis. Table 4. The SNPs and their corresponding phenotypes considered as confounders. [file 41927_2021_216_MOESM1_ESM.docx]

Title: Causal Relationship between Educational Attainment and the Risk of Rheumatoid Arthritis: a Mendelian Randomization Study

Guiwu Huang^1*^, Jiahao Cai^2*^, Wenchang Li^1^, Yanlin Zhong^1^, Weiming Liao^1^, Peihui Wu^1#^

^1^ Department of Joint Surgery, The First Affiliated Hospital of Sun Yat-sen University, Sun Yat-sen University, Guangzhou, China.

^2^ Department of Neurology, Sun Yat-sen Memorial Hospital, Sun Yat-sen University, Guangzhou, China.

^*^equal contribution

^#^Corresponding author: Peihui Wu, Department of Joint Surgery, The First Affiliated Hospital of Sun Yat-sen University, Sun Yat-sen University, Guangzhou, China, Email: [wupeihui3@mail.sysu.edu.cn](mailto:wupeihui3@mail.sysu.edu.cn), Telephone: +86-13332807351

**Additional file**

**Table 1**: Detailed information of SNPs Harmonizing

| **SNPs** | **ea** | **oa** | **eaf** | **beta** | **se** | ***p*-val** | **sample size** |
| --- | --- | --- | --- | --- | --- | --- | --- |
|  |  |  |  |  |  |  |  |
| rs77719387 | A | T | 0.0174 | -0.0403 | 0.0058 | 3.78E-12 | 965119 |
| rs10931821 | A | T | 0.4852 | -0.0141 | 0.0014 | 8.65E-24 | 1130053 |
| rs13085461 | C | G | 0.474 | 0.0103 | 0.0014 | 2.40E-13 | 1131881 |
| rs13130765 | C | G | 0.4721 | -0.0091 | 0.0014 | 1.89E-10 | 1105636 |
| rs1320139 | C | G | 0.427 | -0.0149 | 0.0014 | 7.62E-26 | 1131881 |
| rs13402497 | A | T | 0.4902 | -0.008 | 0.0014 | 1.33E-08 | 1130061 |
| rs2039204 | A | T | 0.5296 | 0.0082 | 0.0014 | 5.23E-09 | 1127815 |
| rs2478208 | C | G | 0.5204 | -0.0103 | 0.0014 | 2.89E-13 | 1129371 |
| rs2517086 | C | G | 0.5721 | -0.0088 | 0.0014 | 8.00E-10 | 1116832 |
| rs6969783 | A | T | 0.4705 | -0.0098 | 0.0014 | 2.87E-12 | 1129624 |
| rs7040995 | C | G | 0.5324 | 0.0097 | 0.0014 | 5.39E-12 | 1131084 |
| rs737945 | C | G | 0.4442 | -0.0115 | 0.0014 | 3.73E-16 | 1131881 |
| rs7920624 | A | T | 0.4783 | 0.012 | 0.0014 | 1.06E-17 | 1131084 |
| rs891793 | C | G | 0.5403 | -0.0099 | 0.0014 | 1.87E-12 | 1128612 |

SNP: single nucleotide polymorphism; ea: effect allele; oa: other allele, eaf: effect allele frequency; beta: β coefficient; se: standard error;

**Table 2:** Detailed information of LD-independent SNPs chosen as instrumental variables for educational attainment (exp) and rheumatoid arthritis (out).

| **SNPs** | **ea** | **oa** | **eaf.exp** | **beta.exp** | **se.exp** | **p-val.exp** | **eaf.out** | **beta.out** | **se.out** | **p-val.out** |
| --- | --- | --- | --- | --- | --- | --- | --- | --- | --- | --- |
| rs62155350 | A | G | 0.0186 | 0.0338 | 0.0053 | 9.01E-11 | 0.014 | 0.1044 | 0.1046 | 0.1592 |
| rs34305371 | A | G | 0.0985 | 0.0314 | 0.0024 | 2.05E-39 | 0.087 | 0 | 0.0408 | 0.5 |
| rs9859556 | T | G | 0.3132 | 0.029 | 0.0015 | 1.41E-83 | 0.336 | 0.0198 | 0.0179 | 0.1337 |
| rs1334297 | A | G | 0.7375 | 0.0257 | 0.0016 | 2.34E-58 | 0.787 | 0.0198 | 0.023 | 0.1942 |
| rs7029718 | A | G | 0.4108 | 0.0244 | 0.0014 | 2.50E-68 | 0.429 | -0.0513 | 0.0179 | 0.002 |
| rs76235882 | A | G | 0.966 | 0.0241 | 0.0039 | 3.22E-10 | 0.973 | 0.0488 | 0.0587 | 0.2028 |
| rs79994730 | T | C | 0.0426 | 0.024 | 0.0035 | 3.51E-12 | 0.037 | -0.0834 | 0.0459 | 0.0347 |
| rs77609760 | A | G | 0.9346 | 0.0236 | 0.0028 | 1.75E-17 | 0.926 | -0.0305 | 0.0306 | 0.1599 |
| rs2436760 | T | C | 0.9717 | 0.0232 | 0.0042 | 1.66E-08 | 0.969 | -0.0305 | 0.0587 | 0.3018 |
| rs9375188 | T | C | 0.4847 | 0.0212 | 0.0014 | 4.22E-52 | 0.508 | -0.0202 | 0.0179 | 0.129 |
| rs11894424 | A | C | 0.9524 | 0.0209 | 0.0033 | 1.20E-10 | 0.942 | -0.0513 | 0.0357 | 0.0755 |
| rs12076635 | C | G | 0.7811 | 0.0207 | 0.0017 | 2.07E-34 | 0.74 | -0.0296 | 0.0179 | 0.0489 |
| rs76076331 | T | C | 0.1346 | 0.0206 | 0.0021 | 5.12E-23 | 0.139 | 0.01 | 0.0332 | 0.3821 |
| rs76577427 | C | G | 0.9006 | 0.0203 | 0.0024 | 1.36E-17 | 0.907 | 0.0202 | 0.0332 | 0.2712 |
| rs176218 | T | G | 0.1958 | 0.02 | 0.0018 | 5.54E-29 | 0.192 | -0.0513 | 0.023 | 0.0127 |
| rs11588857 | A | G | 0.2131 | 0.0199 | 0.0017 | 5.95E-32 | 0.224 | -0.0619 | 0.023 | 0.0035 |
| rs11082011 | T | C | 0.6716 | 0.0194 | 0.0015 | 1.46E-38 | 0.658 | -0.0408 | 0.0204 | 0.0227 |
| rs1689510 | C | G | 0.3315 | 0.0194 | 0.0015 | 1.46E-38 | 0.33 | -0.0488 | 0.0179 | 0.0031 |
| rs10773002 | A | T | 0.252 | 0.0185 | 0.0016 | 3.19E-31 | 0.283 | 0.01 | 0.0179 | 0.2887 |
| rs6449503 | A | G | 0.5038 | 0.0185 | 0.0014 | 3.63E-40 | 0.518 | -0.0101 | 0.0179 | 0.2868 |
| rs11620355 | A | G | 0.0912 | 0.0182 | 0.0025 | 1.67E-13 | 0.11 | -0.0202 | 0.0357 | 0.2858 |
| rs74787922 | A | C | 0.9333 | 0.0182 | 0.0028 | 4.02E-11 | 0.924 | 0 | 0.0383 | 0.5 |
| rs13240401 | T | C | 0.7812 | 0.0176 | 0.0017 | 2.03E-25 | 0.774 | -0.0513 | 0.023 | 0.0127 |
| rs74415461 | T | C | 0.0843 | 0.0176 | 0.0026 | 6.47E-12 | 0.059 | -0.0305 | 0.0434 | 0.2412 |
| rs10875121 | C | G | 0.8361 | 0.0174 | 0.0019 | 2.65E-20 | 0.84 | 0.0408 | 0.0204 | 0.0227 |
| rs73344830 | A | G | 0.4181 | 0.017 | 0.0014 | 3.13E-34 | 0.399 | -0.0305 | 0.0204 | 0.0678 |
| rs4787457 | A | G | 0.6339 | 0.0162 | 0.0015 | 1.72E-27 | 0.684 | -0.0408 | 0.0153 | 0.0038 |
| rs12602286 | T | G | 0.8731 | 0.016 | 0.0021 | 1.28E-14 | 0.885 | -0.0619 | 0.0306 | 0.0216 |
| rs10189857 | A | G | 0.5654 | 0.0158 | 0.0014 | 7.72E-30 | 0.58 | -0.0305 | 0.0179 | 0.044 |
| rs111852224 | T | C | 0.1227 | 0.0156 | 0.0022 | 6.66E-13 | 0.15 | -0.0101 | 0.0306 | 0.3713 |
| rs113520408 | A | G | 0.2756 | 0.0155 | 0.0016 | 1.70E-22 | 0.277 | 0 | 0.023 | 0.5 |
| rs3897821 | A | G | 0.6654 | 0.0154 | 0.0015 | 4.98E-25 | 0.642 | -0.0513 | 0.0179 | 0.002 |
| rs12761761 | T | C | 0.2395 | 0.0153 | 0.0017 | 1.13E-19 | 0.21 | 0.0198 | 0.023 | 0.1942 |
| rs162445 | A | G | 0.0788 | 0.0152 | 0.0026 | 2.52E-09 | 0.094 | 0.0296 | 0.0306 | 0.1671 |
| rs11121177 | A | G | 0.1785 | 0.0151 | 0.0018 | 2.45E-17 | 0.195 | -0.0513 | 0.0255 | 0.0222 |
| rs12571549 | A | G | 0.1368 | 0.0151 | 0.002 | 2.18E-14 | 0.138 | -0.0408 | 0.023 | 0.0377 |
| rs56391344 | A | G | 0.2526 | 0.0148 | 0.0016 | 1.12E-20 | 0.219 | 0 | 0.023 | 0.5 |
| rs12957463 | A | G | 0.7953 | 0.0146 | 0.0018 | 2.51E-16 | 0.826 | -0.0101 | 0.023 | 0.3308 |
| rs178183 | T | C | 0.2455 | 0.0145 | 0.0016 | 6.37E-20 | 0.25 | 0 | 0.023 | 0.5 |
| rs9411331 | A | C | 0.684 | 0.0145 | 0.0015 | 2.09E-22 | 0.689 | -0.0305 | 0.0204 | 0.0678 |
| rs2764684 | T | C | 0.8266 | 0.0143 | 0.0019 | 2.61E-14 | 0.835 | -0.0408 | 0.0255 | 0.0548 |
| rs10772644 | C | G | 0.8887 | 0.0142 | 0.0022 | 5.43E-11 | 0.986 | -0.01 | 0.0306 | 0.3726 |
| rs4766424 | C | G | 0.1237 | 0.0142 | 0.0021 | 6.81E-12 | 0.092 | 0.0202 | 0.0306 | 0.2546 |
| rs17551064 | A | G | 0.8378 | 0.0141 | 0.0019 | 5.81E-14 | 0.84 | -0.0101 | 0.0255 | 0.3468 |
| rs76878669 | C | G | 0.7609 | 0.0141 | 0.0017 | 5.47E-17 | 0.742 | -0.0488 | 0.0255 | 0.0279 |
| rs17411339 | A | G | 0.5612 | 0.014 | 0.0014 | 7.62E-24 | 0.599 | -0.0101 | 0.0204 | 0.3112 |
| rs7683416 | T | C | 0.4592 | 0.0139 | 0.0014 | 1.56E-23 | 0.434 | -0.0101 | 0.0179 | 0.2868 |
| rs3026996 | A | C | 0.7606 | 0.0138 | 0.0016 | 3.20E-18 | 0.737 | -0.0202 | 0.0204 | 0.1611 |
| rs12468040 | T | G | 0.3819 | 0.0137 | 0.0014 | 6.48E-23 | 0.397 | 0 | 0.0204 | 0.5 |
| rs17428076 | C | G | 0.7598 | 0.0137 | 0.0016 | 5.52E-18 | 0.758 | -0.0488 | 0.023 | 0.0168 |
| rs62182994 | T | C | 0.6894 | 0.0137 | 0.0015 | 3.32E-20 | 0.726 | 0 | 0.023 | 0.5 |
| rs7603132 | A | G | 0.1938 | 0.0137 | 0.0018 | 1.36E-14 | 0.172 | -0.0202 | 0.0255 | 0.2142 |
| rs12332731 | A | T | 0.1882 | 0.0135 | 0.0018 | 3.19E-14 | 0.195 | -0.0101 | 0.023 | 0.3308 |
| rs746839 | C | G | 0.6283 | 0.0135 | 0.0015 | 1.13E-19 | 0.659 | -0.0392 | 0.0204 | 0.0273 |
| rs4757957 | C | G | 0.6911 | 0.0133 | 0.0015 | 3.77E-19 | 0.692 | -0.0198 | 0.0204 | 0.1659 |
| rs575113 | A | G | 0.2941 | 0.0133 | 0.0015 | 3.77E-19 | 0.287 | 0 | 0.0204 | 0.5 |
| rs7924036 | T | G | 0.5124 | 0.0133 | 0.0014 | 1.05E-21 | 0.508 | 0.0198 | 0.0153 | 0.0979 |
| rs2838006 | T | C | 0.3597 | 0.0132 | 0.0015 | 6.84E-19 | 0.367 | -0.0101 | 0.0204 | 0.3112 |
| rs12643771 | T | C | 0.3094 | 0.013 | 0.0015 | 2.22E-18 | 0.306 | -0.0202 | 0.0204 | 0.1611 |
| rs6123924 | A | G | 0.8445 | 0.0129 | 0.0019 | 5.63E-12 | 0.842 | -0.0101 | 0.0281 | 0.3601 |
| rs11871429 | A | G | 0.7727 | 0.0128 | 0.0017 | 2.55E-14 | 0.793 | -0.0101 | 0.0204 | 0.3112 |
| rs137079 | T | C | 0.1375 | 0.0128 | 0.002 | 7.77E-11 | 0.114 | 0.0392 | 0.0281 | 0.0811 |
| rs34807077 | A | C | 0.1569 | 0.0128 | 0.0019 | 8.09E-12 | 0.164 | 0.01 | 0.0281 | 0.3614 |
| rs628993 | A | G | 0.1073 | 0.0128 | 0.0023 | 1.31E-08 | 0.094 | 0.0488 | 0.0561 | 0.1923 |
| rs10046069 | A | G | 0.1203 | 0.0127 | 0.0022 | 3.90E-09 | 0.132 | -0.0408 | 0.0281 | 0.0729 |
| rs17489649 | A | G | 0.6788 | 0.0127 | 0.0015 | 1.26E-17 | 0.658 | 0.01 | 0.0204 | 0.3129 |
| rs17604349 | A | G | 0.1852 | 0.0127 | 0.0018 | 8.60E-13 | 0.173 | 0.01 | 0.0255 | 0.3482 |
| rs6917154 | T | C | 0.1236 | 0.0127 | 0.0021 | 7.35E-10 | 0.126 | 0 | 0.0306 | 0.5 |
| rs72993796 | T | C | 0.8836 | 0.0127 | 0.0022 | 3.90E-09 | 0.883 | -0.0726 | 0.0357 | 0.0211 |
| rs9529146 | T | C | 0.2282 | 0.0127 | 0.0017 | 3.99E-14 | 0.224 | -0.0305 | 0.023 | 0.0923 |
| rs11081529 | T | C | 0.7112 | 0.0126 | 0.0015 | 2.23E-17 | 0.75 | -0.0408 | 0.023 | 0.0377 |
| rs2545795 | A | C | 0.4437 | 0.0125 | 0.0014 | 2.16E-19 | 0.472 | -0.0202 | 0.0153 | 0.0934 |
| rs4369924 | A | G | 0.1615 | 0.0125 | 0.0019 | 2.37E-11 | 0.162 | 0.0198 | 0.0281 | 0.2402 |
| rs10080647 | A | C | 0.1405 | 0.0124 | 0.002 | 2.82E-10 | 0.129 | -0.0943 | 0.0255 | 0.0001 |
| rs3859523 | T | C | 0.8208 | 0.0124 | 0.0018 | 2.81E-12 | 0.834 | 0.01 | 0.0255 | 0.3482 |
| rs9882532 | T | C | 0.6368 | 0.0124 | 0.0015 | 6.89E-17 | 0.649 | -0.0305 | 0.0179 | 0.044 |
| rs2081652 | A | T | 0.661 | 0.0123 | 0.0015 | 1.20E-16 | 0.663 | -0.0101 | 0.0204 | 0.3112 |
| rs13261773 | C | G | 0.1602 | 0.0122 | 0.0019 | 6.77E-11 | 0.196 | -0.0198 | 0.0281 | 0.2402 |
| rs1865955 | T | C | 0.8244 | 0.0122 | 0.0019 | 6.77E-11 | 0.813 | 0.0198 | 0.0204 | 0.1659 |
| rs4726070 | A | G | 0.6021 | 0.0122 | 0.0014 | 1.46E-18 | 0.584 | -0.0101 | 0.0204 | 0.3112 |
| rs56794817 | A | G | 0.1615 | 0.0122 | 0.0019 | 6.77E-11 | 0.152 | -0.0305 | 0.0281 | 0.1389 |
| rs7910403 | T | G | 0.1952 | 0.0122 | 0.0018 | 6.10E-12 | 0.196 | -0.0101 | 0.0255 | 0.3468 |
| rs2496482 | T | C | 0.4161 | 0.0121 | 0.0015 | 3.61E-16 | 0.414 | -0.0101 | 0.0153 | 0.2557 |
| rs4467547 | T | G | 0.407 | 0.0121 | 0.0014 | 2.74E-18 | 0.403 | -0.0513 | 0.0179 | 0.002 |
| rs62379838 | T | C | 0.6953 | 0.0121 | 0.0015 | 3.61E-16 | 0.693 | 0.0296 | 0.023 | 0.099 |
| rs12888615 | T | C | 0.1991 | 0.012 | 0.0018 | 1.31E-11 | 0.184 | 0.01 | 0.023 | 0.3324 |
| rs2923424 | A | G | 0.6069 | 0.012 | 0.0014 | 5.11E-18 | 0.602 | 0.01 | 0.0153 | 0.2578 |
| rs56099375 | T | C | 0.241 | 0.012 | 0.0017 | 8.40E-13 | 0.234 | -0.0101 | 0.0255 | 0.3468 |
| rs9933256 | A | G | 0.5558 | 0.0119 | 0.0014 | 9.48E-18 | 0.59 | 0.0198 | 0.0179 | 0.1337 |
| rs143743568 | A | G | 0.154 | 0.0118 | 0.002 | 1.82E-09 | 0.134 | 0.0296 | 0.0383 | 0.2199 |
| rs7974852 | A | C | 0.5235 | 0.0117 | 0.0014 | 3.21E-17 | 0.548 | 0.01 | 0.0204 | 0.3129 |
| rs4915735 | A | G | 0.1408 | 0.0116 | 0.002 | 3.32E-09 | 0.135 | 0.0392 | 0.0306 | 0.1001 |
| rs4839155 | T | G | 0.7671 | 0.0115 | 0.0017 | 6.68E-12 | 0.774 | -0.0619 | 0.023 | 0.0035 |
| rs12967010 | T | C | 0.7746 | 0.0114 | 0.0017 | 1.00E-11 | 0.741 | 0.0198 | 0.0255 | 0.2188 |
| rs7321274 | A | G | 0.7999 | 0.0114 | 0.0018 | 1.20E-10 | 0.806 | 0.01 | 0.0255 | 0.3482 |
| rs10761251 | A | T | 0.6633 | 0.0113 | 0.0015 | 2.47E-14 | 0.61 | 0 | 0.0179 | 0.5 |
| rs10996167 | C | G | 0.6512 | 0.0113 | 0.002 | 8.02E-09 | 0.653 | 0 | 0.0204 | 0.5 |
| rs11542663 | A | C | 0.6947 | 0.0112 | 0.0015 | 4.11E-14 | 0.628 | 0.0296 | 0.0204 | 0.0738 |
| rs2052285 | A | G | 0.5959 | 0.0112 | 0.0014 | 6.22E-16 | 0.561 | -0.0101 | 0.023 | 0.3308 |
| rs62179650 | A | G | 0.2995 | 0.0112 | 0.0016 | 1.28E-12 | 0.273 | 0.01 | 0.0204 | 0.3129 |
| rs743316 | T | C | 0.7915 | 0.0112 | 0.0017 | 2.23E-11 | 0.799 | 0 | 0.0255 | 0.5 |
| rs10940921 | T | G | 0.4194 | 0.0111 | 0.0014 | 1.11E-15 | 0.42 | -0.0101 | 0.0179 | 0.2868 |
| rs73055556 | A | G | 0.1385 | 0.0111 | 0.002 | 1.43E-08 | 0.143 | 0.0677 | 0.0357 | 0.0291 |
| rs10060023 | T | C | 0.3276 | 0.011 | 0.0015 | 1.12E-13 | 0.346 | -0.0101 | 0.023 | 0.3308 |
| rs6065784 | C | G | 0.6953 | 0.011 | 0.0015 | 1.12E-13 | 0.679 | -0.0198 | 0.0204 | 0.1659 |
| rs61527214 | A | G | 0.4113 | 0.011 | 0.0014 | 1.96E-15 | 0.417 | 0.0198 | 0.0153 | 0.0979 |
| rs7972246 | T | C | 0.3451 | 0.011 | 0.0015 | 1.12E-13 | 0.347 | 0 | 0.0204 | 0.5 |
| rs10892807 | T | C | 0.5671 | 0.0109 | 0.0014 | 3.47E-15 | 0.583 | -0.0101 | 0.0204 | 0.3112 |
| rs13197257 | T | G | 0.279 | 0.0109 | 0.0016 | 4.80E-12 | 0.283 | 0.0198 | 0.0179 | 0.1337 |
| rs4320563 | A | G | 0.5093 | 0.0109 | 0.0014 | 3.47E-15 | 0.509 | -0.0305 | 0.0179 | 0.044 |
| rs17048855 | A | G | 0.3426 | 0.0108 | 0.0015 | 3.01E-13 | 0.347 | 0.01 | 0.023 | 0.3324 |
| rs1866823 | A | G | 0.5444 | 0.0107 | 0.0014 | 1.06E-14 | 0.573 | -0.0202 | 0.0204 | 0.1611 |
| rs6924023 | A | G | 0.7548 | 0.0107 | 0.0016 | 1.14E-11 | 0.715 | -0.0202 | 0.0179 | 0.129 |
| rs7575637 | A | G | 0.4538 | 0.0107 | 0.0014 | 1.06E-14 | 0.478 | 0 | 0.0179 | 0.5 |
| rs1747714 | T | C | 0.5287 | 0.0106 | 0.0014 | 1.85E-14 | 0.497 | 0 | 0.0204 | 0.5 |
| rs2256965 | A | G | 0.4188 | 0.0106 | 0.0014 | 1.85E-14 | 0.353 | -0.1744 | 0.0128 | 0 |
| rs6812533 | T | C | 0.7444 | 0.0106 | 0.0016 | 1.74E-11 | 0.746 | 0 | 0.023 | 0.5 |
| rs11138947 | T | C | 0.7236 | 0.0105 | 0.0016 | 2.65E-11 | 0.733 | -0.0305 | 0.0204 | 0.0678 |
| rs35606437 | A | G | 0.2675 | 0.0105 | 0.0016 | 2.65E-11 | 0.312 | -0.0101 | 0.023 | 0.3308 |
| rs10844179 | A | G | 0.2257 | 0.0104 | 0.0017 | 4.75E-10 | 0.238 | -0.0101 | 0.023 | 0.3308 |
| rs11211123 | A | G | 0.2228 | 0.0104 | 0.0017 | 4.75E-10 | 0.218 | -0.0408 | 0.023 | 0.0377 |
| rs1167827 | A | G | 0.4347 | 0.0104 | 0.0014 | 5.49E-14 | 0.46 | 0.01 | 0.0179 | 0.2887 |
| rs4298514 | T | C | 0.6986 | 0.0104 | 0.0015 | 2.06E-12 | 0.708 | -0.0202 | 0.0204 | 0.1611 |
| rs4663617 | A | T | 0.235 | 0.0104 | 0.0017 | 4.75E-10 | 0.21 | -0.0619 | 0.023 | 0.0035 |
| rs2199409 | T | C | 0.7962 | 0.0103 | 0.0017 | 6.86E-10 | 0.809 | -0.0101 | 0.0255 | 0.3468 |
| rs12524795 | T | C | 0.4374 | 0.0102 | 0.0014 | 1.60E-13 | 0.437 | 0.0198 | 0.023 | 0.1942 |
| rs4384309 | A | G | 0.4641 | 0.0102 | 0.0014 | 1.60E-13 | 0.467 | 0 | 0.0204 | 0.5 |
| rs6994287 | A | G | 0.4055 | 0.0102 | 0.0014 | 1.60E-13 | 0.412 | 0.0296 | 0.0204 | 0.0738 |
| rs7449561 | A | G | 0.226 | 0.0102 | 0.0017 | 9.87E-10 | 0.25 | 0 | 0.0255 | 0.5 |
| rs11030102 | C | G | 0.7479 | 0.0101 | 0.0016 | 1.37E-10 | 0.766 | -0.0296 | 0.023 | 0.099 |
| rs13212041 | T | C | 0.7978 | 0.0101 | 0.0018 | 1.01E-08 | 0.811 | 0 | 0.0255 | 0.5 |
| rs17148998 | A | G | 0.2042 | 0.0101 | 0.0017 | 1.41E-09 | 0.197 | 0 | 0.023 | 0.5 |
| rs730384 | A | G | 0.4388 | 0.0101 | 0.0014 | 2.71E-13 | 0.437 | -0.0305 | 0.0179 | 0.044 |
| rs806816 | A | T | 0.7498 | 0.0101 | 0.0016 | 1.37E-10 | 0.768 | 0.01 | 0.023 | 0.3324 |
| rs1933264 | T | C | 0.7491 | 0.01 | 0.0016 | 2.05E-10 | 0.746 | -0.0202 | 0.023 | 0.1894 |
| rs590013 | T | C | 0.6772 | 0.01 | 0.0015 | 1.31E-11 | 0.638 | -0.0305 | 0.0204 | 0.0678 |
| rs2297293 | C | G | 0.3141 | 0.0099 | 0.0015 | 2.06E-11 | 0.309 | 0.0305 | 0.0204 | 0.0678 |
| rs17144467 | A | C | 0.6867 | 0.0098 | 0.0015 | 3.22E-11 | 0.698 | -0.0101 | 0.0179 | 0.2868 |
| rs2216144 | T | C | 0.5306 | 0.0098 | 0.0014 | 1.28E-12 | 0.546 | -0.0408 | 0.0179 | 0.0111 |
| rs4846724 | A | G | 0.5347 | 0.0098 | 0.0014 | 1.28E-12 | 0.527 | 0 | 0.0179 | 0.5 |
| rs10193498 | A | T | 0.755 | 0.0097 | 0.0016 | 6.70E-10 | 0.735 | -0.0513 | 0.023 | 0.0127 |
| rs12054166 | C | G | 0.7399 | 0.0096 | 0.0016 | 9.87E-10 | 0.738 | -0.0198 | 0.023 | 0.1942 |
| rs13133213 | A | G | 0.498 | 0.0096 | 0.0014 | 3.51E-12 | 0.495 | -0.0305 | 0.0179 | 0.044 |
| rs1426619 | T | C | 0.4346 | 0.0096 | 0.0014 | 3.51E-12 | 0.447 | 0 | 0.0153 | 0.5 |
| rs2447097 | T | G | 0.4596 | 0.0096 | 0.0014 | 3.51E-12 | 0.475 | -0.0408 | 0.0179 | 0.0111 |
| rs2469226 | A | T | 0.2228 | 0.0096 | 0.0017 | 8.16E-09 | 0.206 | 0.0296 | 0.0255 | 0.1233 |
| rs4328757 | T | C | 0.6119 | 0.0096 | 0.0014 | 3.51E-12 | 0.624 | -0.0202 | 0.0204 | 0.1611 |
| rs9929762 | A | G | 0.5595 | 0.0096 | 0.0014 | 3.51E-12 | 0.57 | -0.0101 | 0.0204 | 0.3112 |
| rs252991 | A | G | 0.3695 | 0.0095 | 0.0015 | 1.20E-10 | 0.366 | 0 | 0.0204 | 0.5 |
| rs11724690 | T | G | 0.2887 | 0.0093 | 0.0015 | 2.82E-10 | 0.312 | 0.01 | 0.023 | 0.3324 |
| rs12438177 | A | G | 0.3676 | 0.0093 | 0.0015 | 2.82E-10 | 0.384 | 0.01 | 0.0204 | 0.3129 |
| rs4895650 | T | C | 0.5472 | 0.0093 | 0.0014 | 1.54E-11 | 0.53 | -0.0202 | 0.0204 | 0.1611 |
| rs57349798 | A | G | 0.4094 | 0.0093 | 0.0014 | 1.54E-11 | 0.434 | 0 | 0.0204 | 0.5 |
| rs10830858 | T | C | 0.5104 | 0.0092 | 0.0014 | 2.49E-11 | 0.513 | -0.0408 | 0.0204 | 0.0227 |
| rs12670376 | A | G | 0.4464 | 0.0092 | 0.0014 | 2.49E-11 | 0.459 | 0 | 0.0204 | 0.5 |
| rs12789313 | T | C | 0.4907 | 0.0092 | 0.0014 | 2.49E-11 | 0.517 | 0.01 | 0.0204 | 0.3129 |
| rs7012546 | T | C | 0.4166 | 0.0092 | 0.0014 | 2.49E-11 | 0.417 | -0.0305 | 0.0204 | 0.0678 |
| rs11003463 | T | G | 0.6114 | 0.0091 | 0.0015 | 6.53E-10 | 0.621 | 0 | 0.0255 | 0.5 |
| rs11023764 | A | G | 0.6418 | 0.0091 | 0.0015 | 6.53E-10 | 0.675 | 0.0198 | 0.0204 | 0.1659 |
| rs2434672 | A | C | 0.5321 | 0.0091 | 0.0014 | 4.02E-11 | 0.529 | 0.01 | 0.0204 | 0.3129 |
| rs9633970 | T | C | 0.7606 | 0.0091 | 0.0016 | 6.45E-09 | 0.753 | 0.01 | 0.023 | 0.3324 |
| rs11130380 | T | G | 0.6135 | 0.009 | 0.0014 | 6.44E-11 | 0.609 | -0.0101 | 0.0179 | 0.2868 |
| rs1603460 | T | G | 0.4206 | 0.009 | 0.0014 | 6.44E-11 | 0.417 | -0.0202 | 0.0204 | 0.1611 |
| rs232496 | T | C | 0.3657 | 0.009 | 0.0015 | 9.87E-10 | 0.403 | 0.01 | 0.0204 | 0.3129 |
| rs2034631 | T | C | 0.6508 | 0.0089 | 0.0015 | 1.48E-09 | 0.654 | -0.0202 | 0.0204 | 0.1611 |
| rs12113634 | T | C | 0.6504 | 0.0088 | 0.0015 | 2.22E-09 | 0.664 | 0.01 | 0.0204 | 0.3129 |
| rs1564347 | T | G | 0.3433 | 0.0088 | 0.0015 | 2.22E-09 | 0.362 | 0.01 | 0.0204 | 0.3129 |
| rs2554835 | A | G | 0.3998 | 0.0088 | 0.0014 | 1.63E-10 | 0.403 | -0.0101 | 0.0204 | 0.3112 |
| rs2885198 | A | G | 0.5318 | 0.0088 | 0.0014 | 1.63E-10 | 0.486 | -0.0101 | 0.0204 | 0.3112 |
| rs2964255 | A | G | 0.3066 | 0.0088 | 0.0015 | 2.22E-09 | 0.289 | -0.0202 | 0.0204 | 0.1611 |
| rs6490618 | T | C | 0.327 | 0.0088 | 0.0015 | 2.22E-09 | 0.328 | -0.0513 | 0.0204 | 0.006 |
| rs912883 | T | C | 0.6781 | 0.0087 | 0.0015 | 3.32E-09 | 0.642 | -0.0101 | 0.0204 | 0.3112 |
| rs11076962 | T | C | 0.7189 | 0.0086 | 0.0016 | 3.83E-08 | 0.716 | -0.0101 | 0.0153 | 0.2557 |
| rs2007655 | T | G | 0.4976 | 0.0086 | 0.0014 | 4.05E-10 | 0.519 | -0.0101 | 0.0204 | 0.3112 |
| rs42302 | A | G | 0.3441 | 0.0086 | 0.0015 | 4.92E-09 | 0.347 | -0.0202 | 0.0204 | 0.1611 |
| rs736281 | T | C | 0.3931 | 0.0086 | 0.0014 | 4.05E-10 | 0.366 | 0.01 | 0.0204 | 0.3129 |
| rs8097125 | T | C | 0.3802 | 0.0086 | 0.0014 | 4.05E-10 | 0.372 | -0.0101 | 0.0204 | 0.3112 |
| rs72709560 | A | G | 0.6865 | 0.0085 | 0.0015 | 7.28E-09 | 0.695 | 0 | 0.0204 | 0.5 |
| rs7894722 | T | C | 0.3748 | 0.0085 | 0.0014 | 6.34E-10 | 0.371 | -0.0101 | 0.0204 | 0.3112 |
| rs8030487 | A | G | 0.6907 | 0.0085 | 0.0015 | 7.28E-09 | 0.661 | 0 | 0.0204 | 0.5 |
| rs1505676 | C | G | 0.6293 | 0.0084 | 0.0015 | 1.07E-08 | 0.67 | 0.0202 | 0.0179 | 0.129 |
| rs17882802 | A | G | 0.4295 | 0.0084 | 0.0014 | 9.87E-10 | 0.427 | -0.0101 | 0.0204 | 0.3112 |
| rs2183271 | T | C | 0.6364 | 0.0084 | 0.0015 | 1.07E-08 | 0.618 | 0.0198 | 0.0204 | 0.1659 |
| rs4793090 | A | G | 0.6635 | 0.0084 | 0.0015 | 1.07E-08 | 0.677 | -0.0101 | 0.0179 | 0.2868 |
| rs12614263 | A | G | 0.4959 | 0.0083 | 0.0014 | 1.53E-09 | 0.479 | -0.0101 | 0.0204 | 0.3112 |
| rs2055940 | A | G | 0.3215 | 0.0082 | 0.0015 | 2.29E-08 | 0.327 | 0.0296 | 0.0179 | 0.0489 |
| rs2958182 | A | T | 0.3414 | 0.0082 | 0.0015 | 2.29E-08 | 0.328 | -0.0305 | 0.0204 | 0.0678 |
| rs6977237 | T | C | 0.4725 | 0.0082 | 0.0014 | 2.35E-09 | 0.457 | -0.0408 | 0.0179 | 0.0111 |
| rs9927137 | A | G | 0.5298 | 0.0082 | 0.0014 | 2.35E-09 | 0.515 | -0.0513 | 0.0153 | 0.0004 |
| rs2131167 | A | G | 0.4458 | 0.008 | 0.0014 | 5.51E-09 | 0.433 | -0.0202 | 0.0204 | 0.1611 |
| rs9492774 | C | G | 0.3692 | 0.008 | 0.0015 | 4.82E-08 | 0.375 | 0 | 0.0204 | 0.5 |
| rs2989476 | C | G | 0.4193 | 0.0078 | 0.0014 | 1.26E-08 | 0.39 | -0.0198 | 0.0153 | 0.0979 |
| rs7226824 | C | T | 0.4695 | 0.0078 | 0.0014 | 1.26E-08 | 0.491 | 0.01 | 0.0204 | 0.3129 |
| rs7875078 | C | A | 0.5436 | 0.0078 | 0.0014 | 1.26E-08 | 0.476 | 0.0392 | 0.0204 | 0.0273 |
| rs1220779 | G | A | 0.5321 | 0.0079 | 0.0014 | 8.36E-09 | 0.452 | 0 | 0.0204 | 0.5 |
| rs936496 | G | A | 0.3771 | 0.0079 | 0.0014 | 8.36E-09 | 0.621 | -0.0305 | 0.0179 | 0.044 |
| rs12574281 | C | A | 0.369 | 0.008 | 0.0015 | 4.82E-08 | 0.636 | 0.01 | 0.0204 | 0.3129 |
| rs13050131 | C | A | 0.3353 | 0.0081 | 0.0015 | 3.33E-08 | 0.639 | 0.0198 | 0.0204 | 0.1659 |
| rs1861786 | G | A | 0.6187 | 0.0081 | 0.0014 | 3.61E-09 | 0.371 | 0.01 | 0.0153 | 0.2578 |
| rs2929032 | G | A | 0.5346 | 0.0081 | 0.0014 | 3.61E-09 | 0.472 | -0.0101 | 0.0179 | 0.2868 |
| rs173003 | C | A | 0.5066 | 0.0082 | 0.0014 | 2.35E-09 | 0.495 | 0.0392 | 0.0153 | 0.0052 |
| rs382196 | G | T | 0.6186 | 0.0082 | 0.0014 | 2.35E-09 | 0.338 | 0.0296 | 0.0204 | 0.0738 |
| rs4904523 | G | A | 0.4792 | 0.0082 | 0.0014 | 2.35E-09 | 0.483 | 0.01 | 0.0204 | 0.3129 |
| rs6043521 | C | T | 0.3965 | 0.0083 | 0.0014 | 1.53E-09 | 0.604 | 0.01 | 0.0204 | 0.3129 |
| rs1542354 | G | A | 0.5236 | 0.0084 | 0.0014 | 9.87E-10 | 0.474 | -0.0101 | 0.0204 | 0.3112 |
| rs4977885 | G | A | 0.6029 | 0.0084 | 0.0014 | 9.87E-10 | 0.41 | 0 | 0.0204 | 0.5 |
| rs13266287 | T | A | 0.3313 | 0.0085 | 0.0015 | 7.28E-09 | 0.633 | 0.0198 | 0.0204 | 0.1659 |
| rs4810894 | G | A | 0.6294 | 0.0085 | 0.0015 | 7.28E-09 | 0.378 | 0.0392 | 0.0204 | 0.0273 |
| rs66721975 | G | A | 0.7097 | 0.0087 | 0.0015 | 3.32E-09 | 0.282 | -0.0619 | 0.0204 | 0.0012 |
| rs12765185 | T | A | 0.7231 | 0.0088 | 0.0016 | 1.90E-08 | 0.282 | 0.01 | 0.0281 | 0.3614 |
| rs7672622 | G | A | 0.2541 | 0.0088 | 0.0016 | 1.90E-08 | 0.725 | 0.01 | 0.023 | 0.3324 |
| rs10799615 | G | A | 0.7475 | 0.0089 | 0.0016 | 1.33E-08 | 0.241 | 0.0198 | 0.023 | 0.1942 |
| rs1890132 | C | T | 0.2944 | 0.0089 | 0.0015 | 1.48E-09 | 0.663 | 0.01 | 0.0204 | 0.3129 |
| rs3948495 | G | T | 0.5921 | 0.0089 | 0.0014 | 1.03E-10 | 0.403 | 0 | 0.0204 | 0.5 |
| rs7650602 | C | T | 0.4401 | 0.0089 | 0.0014 | 1.03E-10 | 0.577 | -0.0305 | 0.0153 | 0.0233 |
| rs3111251 | C | T | 0.5675 | 0.009 | 0.0014 | 6.44E-11 | 0.445 | 0.0488 | 0.0204 | 0.0084 |
| rs11657979 | G | A | 0.7551 | 0.0091 | 0.0017 | 4.33E-08 | 0.225 | 0.0583 | 0.0357 | 0.0514 |
| rs2011603 | G | A | 0.2641 | 0.0091 | 0.0016 | 6.45E-09 | 0.707 | 0 | 0.023 | 0.5 |
| rs1747817 | C | T | 0.2386 | 0.0092 | 0.0016 | 4.46E-09 | 0.769 | 0.0392 | 0.0204 | 0.0273 |
| rs4778058 | C | T | 0.5036 | 0.0092 | 0.0014 | 2.49E-11 | 0.478 | -0.0101 | 0.0179 | 0.2868 |
| rs10006235 | C | T | 0.7311 | 0.0093 | 0.0016 | 3.08E-09 | 0.285 | 0.0392 | 0.023 | 0.0438 |
| rs17110109 | C | T | 0.3837 | 0.0093 | 0.0014 | 1.54E-11 | 0.625 | 0.01 | 0.023 | 0.3324 |
| rs1738050 | G | C | 0.3803 | 0.0093 | 0.0014 | 1.54E-11 | 0.598 | 0.0408 | 0.0204 | 0.0227 |
| rs4675248 | G | A | 0.6005 | 0.0094 | 0.0014 | 9.45E-12 | 0.387 | 0.01 | 0.0204 | 0.3129 |
| rs7167688 | C | T | 0.5083 | 0.0094 | 0.0014 | 9.45E-12 | 0.53 | 0.01 | 0.0204 | 0.3129 |
| rs73191311 | G | A | 0.6637 | 0.0094 | 0.0015 | 1.84E-10 | 0.326 | 0.01 | 0.0179 | 0.2887 |
| rs17732878 | C | T | 0.2088 | 0.0095 | 0.0017 | 1.15E-08 | 0.814 | 0 | 0.0204 | 0.5 |
| rs4358081 | C | A | 0.4715 | 0.0095 | 0.0014 | 5.78E-12 | 0.544 | -0.0408 | 0.0204 | 0.0227 |
| rs1301838 | C | T | 0.6773 | 0.0096 | 0.0015 | 7.77E-11 | 0.31 | 0.0198 | 0.0204 | 0.1659 |
| rs1544 | G | A | 0.7364 | 0.0096 | 0.0016 | 9.87E-10 | 0.277 | 0.01 | 0.0204 | 0.3129 |
| rs1329125 | C | T | 0.6727 | 0.0097 | 0.0015 | 5.01E-11 | 0.322 | 0.0198 | 0.0204 | 0.1659 |
| rs3751331 | G | A | 0.6203 | 0.0097 | 0.0014 | 2.13E-12 | 0.372 | -0.0101 | 0.0179 | 0.2868 |
| rs401966 | G | C | 0.61 | 0.0097 | 0.0014 | 2.13E-12 | 0.391 | 0.0305 | 0.0179 | 0.044 |
| rs7617204 | G | A | 0.4777 | 0.0097 | 0.0014 | 2.13E-12 | 0.516 | 0.0198 | 0.0153 | 0.0979 |
| rs42210 | G | C | 0.2878 | 0.0098 | 0.0016 | 4.53E-10 | 0.685 | -0.0296 | 0.023 | 0.099 |
| rs72944064 | C | T | 0.2563 | 0.0098 | 0.0016 | 4.53E-10 | 0.75 | -0.0202 | 0.0204 | 0.1611 |
| rs1245829 | T | A | 0.4186 | 0.0099 | 0.0014 | 7.67E-13 | 0.58 | 0 | 0.0204 | 0.5 |
| rs2964199 | C | T | 0.6851 | 0.0099 | 0.0015 | 2.06E-11 | 0.308 | 0.0296 | 0.023 | 0.099 |
| rs9929556 | G | T | 0.4312 | 0.0099 | 0.0014 | 7.67E-13 | 0.541 | 0.01 | 0.0204 | 0.3129 |
| rs175325 | T | A | 0.4034 | 0.01 | 0.0014 | 4.57E-13 | 0.598 | 0 | 0.0204 | 0.5 |
| rs2212430 | C | T | 0.6962 | 0.01 | 0.0016 | 2.05E-10 | 0.32 | 0 | 0.0153 | 0.5 |
| rs1991585 | C | T | 0.294 | 0.0101 | 0.0015 | 8.29E-12 | 0.695 | -0.0305 | 0.0179 | 0.044 |
| rs7171405 | G | A | 0.7568 | 0.0101 | 0.0016 | 1.37E-10 | 0.263 | 0.0198 | 0.023 | 0.1942 |
| rs72672052 | T | A | 0.8247 | 0.0101 | 0.0018 | 1.01E-08 | 0.171 | 0.0296 | 0.0255 | 0.1233 |
| rs10805383 | G | A | 0.5121 | 0.0103 | 0.0014 | 9.39E-14 | 0.517 | 0.0488 | 0.0204 | 0.0084 |
| rs4899012 | G | C | 0.3981 | 0.0103 | 0.0014 | 9.39E-14 | 0.591 | 0.0408 | 0.0204 | 0.0227 |
| rs2287838 | G | A | 0.4496 | 0.0104 | 0.0014 | 5.49E-14 | 0.542 | 0.01 | 0.0204 | 0.3129 |
| rs34067381 | G | T | 0.6383 | 0.0104 | 0.0015 | 2.06E-12 | 0.425 | 0 | 0.0204 | 0.5 |
| rs401526 | C | T | 0.4742 | 0.0104 | 0.0014 | 5.49E-14 | 0.501 | 0.0198 | 0.0204 | 0.1659 |
| rs11212135* | C | T | 0.7541 | 0.0104 | 0.0016 | 4.02E-11 | 0.269 | 0.01 | 0.023 | 0.3324 |
| rs2364544 | G | A | 0.6088 | 0.0105 | 0.0014 | 3.19E-14 | 0.432 | -0.0202 | 0.0179 | 0.129 |
| rs62174974 | G | A | 0.8051 | 0.0105 | 0.0018 | 2.72E-09 | 0.232 | 0.01 | 0.0255 | 0.3482 |
| rs9359939 | C | A | 0.7569 | 0.0105 | 0.0016 | 2.65E-11 | 0.229 | 0.0198 | 0.023 | 0.1942 |
| rs11213482 | G | A | 0.164 | 0.0106 | 0.0019 | 1.21E-08 | 0.842 | 0.01 | 0.0357 | 0.3903 |
| rs11598765 | G | A | 0.8034 | 0.0106 | 0.0018 | 1.94E-09 | 0.211 | 0 | 0.0281 | 0.5 |
| rs17565975 | G | A | 0.4437 | 0.0106 | 0.0014 | 1.85E-14 | 0.505 | 0 | 0.0204 | 0.5 |
| rs8008382 | C | T | 0.6991 | 0.0106 | 0.0015 | 7.93E-13 | 0.307 | 0 | 0.0255 | 0.5 |
| rs969512 | T | A | 0.3404 | 0.0106 | 0.0015 | 7.93E-13 | 0.695 | -0.0202 | 0.0204 | 0.1611 |
| rs12519073 | C | T | 0.7683 | 0.0107 | 0.0017 | 1.55E-10 | 0.239 | 0.01 | 0.023 | 0.3324 |
| rs12638072 | G | A | 0.7008 | 0.0107 | 0.0015 | 4.90E-13 | 0.31 | 0 | 0.0204 | 0.5 |
| rs12875339 | C | A | 0.3551 | 0.0108 | 0.0015 | 3.01E-13 | 0.658 | -0.0202 | 0.0204 | 0.1611 |
| rs10951590 | C | T | 0.6725 | 0.0109 | 0.0015 | 1.84E-13 | 0.308 | 0 | 0.0204 | 0.5 |
| rs3809634 | G | A | 0.3158 | 0.0109 | 0.0015 | 1.84E-13 | 0.692 | 0 | 0.0204 | 0.5 |
| rs4500930 | C | T | 0.6551 | 0.0109 | 0.0015 | 1.84E-13 | 0.34 | 0.0296 | 0.0204 | 0.0738 |
| rs7692359 | C | T | 0.2214 | 0.0109 | 0.0017 | 7.19E-11 | 0.785 | 0.01 | 0.023 | 0.3324 |
| rs10773208 | C | T | 0.7474 | 0.011 | 0.0016 | 3.10E-12 | 0.248 | -0.0101 | 0.0179 | 0.2868 |
| rs13422673 | C | T | 0.5341 | 0.011 | 0.0014 | 1.96E-15 | 0.503 | 0.01 | 0.0204 | 0.3129 |
| rs28513882 | G | A | 0.8217 | 0.011 | 0.0018 | 4.95E-10 | 0.172 | 0.01 | 0.0204 | 0.3129 |
| rs303752 | G | A | 0.5933 | 0.011 | 0.0014 | 1.96E-15 | 0.394 | 0.0488 | 0.023 | 0.0168 |
| rs9556958 | C | T | 0.4767 | 0.011 | 0.0014 | 1.96E-15 | 0.522 | -0.0202 | 0.0179 | 0.129 |
| rs7041702 | G | A | 0.2594 | 0.0111 | 0.0016 | 2.00E-12 | 0.723 | 0.01 | 0.023 | 0.3324 |
| rs9373363 | G | A | 0.2515 | 0.0111 | 0.0016 | 2.00E-12 | 0.75 | 0.01 | 0.023 | 0.3324 |
| rs12981405 | C | T | 0.8336 | 0.0112 | 0.0019 | 1.88E-09 | 0.147 | 0 | 0.0255 | 0.5 |
| rs12503522 | C | T | 0.718 | 0.0113 | 0.0016 | 8.18E-13 | 0.26 | 0.0296 | 0.023 | 0.099 |
| rs488476 | G | C | 0.3809 | 0.0113 | 0.0015 | 2.47E-14 | 0.626 | 0.0305 | 0.0153 | 0.0233 |
| rs6690195 | C | T | 0.5093 | 0.0113 | 0.0014 | 3.47E-16 | 0.472 | 0 | 0.0204 | 0.5 |
| rs35929923 | G | A | 0.7505 | 0.0114 | 0.0016 | 5.20E-13 | 0.239 | -0.0101 | 0.023 | 0.3308 |
| rs57661533 | C | T | 0.8659 | 0.0114 | 0.0021 | 2.84E-08 | 0.149 | 0.0296 | 0.0306 | 0.1671 |
| rs6731373 | G | A | 0.661 | 0.0115 | 0.0015 | 8.83E-15 | 0.299 | 0.0198 | 0.023 | 0.1942 |
| rs8024 | C | A | 0.6706 | 0.0115 | 0.0015 | 8.83E-15 | 0.301 | 0.01 | 0.023 | 0.3324 |
| rs17598675 | C | T | 0.4868 | 0.0116 | 0.0014 | 5.87E-17 | 0.497 | 0.01 | 0.0179 | 0.2887 |
| rs6704768 | G | A | 0.4352 | 0.0116 | 0.0014 | 5.87E-17 | 0.529 | 0.0488 | 0.0204 | 0.0084 |
| rs10979613 | C | T | 0.3611 | 0.0117 | 0.0015 | 3.10E-15 | 0.643 | -0.0101 | 0.0153 | 0.2557 |
| rs1717204 | C | A | 0.8182 | 0.0117 | 0.0018 | 4.02E-11 | 0.18 | 0.0198 | 0.0281 | 0.2402 |
| rs1842713 | G | A | 0.2118 | 0.0117 | 0.0017 | 2.94E-12 | 0.799 | -0.0202 | 0.023 | 0.1894 |
| rs34394051 | G | A | 0.1571 | 0.0117 | 0.002 | 2.46E-09 | 0.832 | 0.0198 | 0.0281 | 0.2402 |
| rs4848924 | C | A | 0.29 | 0.0117 | 0.0015 | 3.10E-15 | 0.702 | 0 | 0.023 | 0.5 |
| rs7254263 | C | T | 0.715 | 0.0119 | 0.0016 | 5.13E-14 | 0.26 | 0 | 0.0204 | 0.5 |
| rs72622559 | C | T | 0.7699 | 0.012 | 0.0017 | 8.40E-13 | 0.253 | 0.01 | 0.023 | 0.3324 |
| rs13327482 | G | A | 0.1779 | 0.0121 | 0.0018 | 8.95E-12 | 0.837 | 0.0392 | 0.0281 | 0.0811 |
| rs2570497 | C | T | 0.3633 | 0.0121 | 0.0015 | 3.61E-16 | 0.674 | 0.0198 | 0.0204 | 0.1659 |
| rs4719944 | C | T | 0.4538 | 0.0121 | 0.0014 | 2.74E-18 | 0.518 | 0.01 | 0.0179 | 0.2887 |
| rs114593137 | T | A | 0.207 | 0.0122 | 0.0017 | 3.58E-13 | 0.781 | 0.0392 | 0.0255 | 0.0621 |
| rs6493265 | C | T | 0.6085 | 0.0122 | 0.0014 | 1.46E-18 | 0.382 | 0.01 | 0.0204 | 0.3129 |
| rs717996 | C | T | 0.4014 | 0.0122 | 0.0014 | 1.46E-18 | 0.601 | -0.0305 | 0.0179 | 0.044 |
| rs113615161 | C | T | 0.867 | 0.0123 | 0.0021 | 2.35E-09 | 0.128 | 0.01 | 0.0332 | 0.3821 |
| rs7460106 | C | T | 0.238 | 0.0123 | 0.0017 | 2.32E-13 | 0.75 | 0.0583 | 0.0306 | 0.0285 |
| rs10460095 | G | A | 0.4286 | 0.0124 | 0.0014 | 4.10E-19 | 0.585 | 0.0392 | 0.023 | 0.0438 |
| rs5754753 | C | T | 0.2822 | 0.0126 | 0.0016 | 1.70E-15 | 0.738 | 0.01 | 0.0204 | 0.3129 |
| rs6697584 | C | T | 0.2148 | 0.0126 | 0.0017 | 6.23E-14 | 0.805 | -0.0101 | 0.023 | 0.3308 |
| rs68145588 | G | T | 0.8605 | 0.0127 | 0.0021 | 7.35E-10 | 0.118 | -0.0101 | 0.0306 | 0.3713 |
| rs9853928 | C | T | 0.7898 | 0.0127 | 0.0017 | 3.99E-14 | 0.247 | 0.0198 | 0.023 | 0.1942 |
| rs7326331 | G | A | 0.7239 | 0.0128 | 0.0016 | 6.22E-16 | 0.254 | 0.0296 | 0.0204 | 0.0738 |
| rs11663602 | C | A | 0.7239 | 0.013 | 0.0016 | 2.24E-16 | 0.25 | -0.0101 | 0.0153 | 0.2557 |
| rs12506221 | G | T | 0.564 | 0.013 | 0.0014 | 8.03E-21 | 0.459 | 0.0296 | 0.0204 | 0.0738 |
| rs6065080 | C | T | 0.6411 | 0.013 | 0.0015 | 2.22E-18 | 0.353 | 0.0198 | 0.0204 | 0.1659 |
| rs9927842 | C | T | 0.851 | 0.013 | 0.002 | 4.02E-11 | 0.152 | -0.0202 | 0.0357 | 0.2858 |
| rs112603734 | C | A | 0.2301 | 0.0131 | 0.0022 | 1.30E-09 | 0.772 | 0.0392 | 0.023 | 0.0438 |
| rs2179152 | C | T | 0.6327 | 0.0131 | 0.0015 | 1.24E-18 | 0.364 | -0.0202 | 0.0153 | 0.0934 |
| rs12273435 | G | A | 0.793 | 0.0132 | 0.0017 | 4.09E-15 | 0.194 | 0.0198 | 0.0306 | 0.2589 |
| rs12646523 | C | T | 0.7517 | 0.0132 | 0.0016 | 7.92E-17 | 0.253 | -0.0202 | 0.0204 | 0.1611 |
| rs79728014 | G | A | 0.1447 | 0.0134 | 0.002 | 1.04E-11 | 0.875 | 0.01 | 0.0306 | 0.3726 |
| rs7977614 | G | A | 0.2857 | 0.0134 | 0.0016 | 2.76E-17 | 0.71 | 0.0296 | 0.0204 | 0.0738 |
| rs9616947 | C | T | 0.6182 | 0.0134 | 0.0015 | 2.07E-19 | 0.388 | 0.0198 | 0.0255 | 0.2188 |
| rs17248751 | G | A | 0.2173 | 0.0136 | 0.0017 | 6.22E-16 | 0.786 | 0.0296 | 0.0255 | 0.1233 |
| rs2998299 | G | T | 0.7863 | 0.0136 | 0.0017 | 6.22E-16 | 0.231 | 0.01 | 0.0255 | 0.3482 |
| rs9886703 | T | A | 0.8329 | 0.0136 | 0.0019 | 4.10E-13 | 0.17 | 0.01 | 0.0255 | 0.3482 |
| rs1434630 | G | T | 0.8521 | 0.0137 | 0.002 | 3.69E-12 | 0.15 | -0.0101 | 0.0255 | 0.3468 |
| rs12375949 | C | T | 0.5688 | 0.0138 | 0.0014 | 3.19E-23 | 0.41 | 0.01 | 0.0179 | 0.2887 |
| rs4673840 | C | T | 0.158 | 0.0138 | 0.0019 | 1.89E-13 | 0.836 | 0 | 0.0281 | 0.5 |
| rs4984541 | G | A | 0.2334 | 0.0138 | 0.0017 | 2.38E-16 | 0.752 | 0.0392 | 0.0332 | 0.1185 |
| rs11157931 | C | A | 0.6076 | 0.0139 | 0.0014 | 1.56E-23 | 0.388 | 0.0392 | 0.0179 | 0.014 |
| rs10798888 | G | T | 0.8275 | 0.014 | 0.0019 | 8.63E-14 | 0.159 | 0.01 | 0.0281 | 0.3614 |
| rs1128956 | G | T | 0.1749 | 0.014 | 0.0019 | 8.63E-14 | 0.808 | 0 | 0.0306 | 0.5 |
| rs1955250 | C | A | 0.0863 | 0.014 | 0.0025 | 1.07E-08 | 0.903 | 0.0296 | 0.0383 | 0.2199 |
| rs77025239 | G | A | 0.8441 | 0.014 | 0.0019 | 8.63E-14 | 0.137 | -0.0101 | 0.0281 | 0.3601 |
| rs117398064 | G | C | 0.9088 | 0.0141 | 0.0025 | 8.50E-09 | 0.086 | 0.0513 | 0.0306 | 0.0469 |
| rs2725370 | C | T | 0.6983 | 0.0141 | 0.0015 | 2.73E-21 | 0.288 | -0.0101 | 0.0204 | 0.3112 |
| rs78648104 | C | T | 0.0863 | 0.0141 | 0.0025 | 8.50E-09 | 0.883 | -0.0305 | 0.0357 | 0.1969 |
| rs363096 | C | T | 0.5755 | 0.0143 | 0.0014 | 8.56E-25 | 0.409 | 0.0198 | 0.0204 | 0.1659 |
| rs61997667 | C | T | 0.8489 | 0.0144 | 0.002 | 3.01E-13 | 0.141 | 0.0392 | 0.0281 | 0.0811 |
| rs790647 | C | A | 0.7722 | 0.0145 | 0.0017 | 7.35E-18 | 0.218 | 0 | 0.023 | 0.5 |
| rs17563464 | C | A | 0.7827 | 0.0147 | 0.0017 | 2.64E-18 | 0.185 | 0 | 0.0281 | 0.5 |
| rs580652 | C | T | 0.0983 | 0.0147 | 0.0024 | 4.53E-10 | 0.916 | -0.0202 | 0.0332 | 0.2712 |
| rs10215082 | G | A | 0.5339 | 0.0149 | 0.0014 | 9.41E-27 | 0.442 | 0.0198 | 0.0204 | 0.1659 |
| rs12151248 | C | T | 0.8859 | 0.0149 | 0.0023 | 4.64E-11 | 0.096 | -0.0619 | 0.0357 | 0.0416 |
| rs13163845 | C | T | 0.1512 | 0.0149 | 0.002 | 4.67E-14 | 0.848 | 0.01 | 0.0306 | 0.3726 |
| rs2290601 | C | T | 0.228 | 0.0149 | 0.0017 | 9.36E-19 | 0.781 | -0.0101 | 0.0204 | 0.3112 |
| rs72829857 | G | A | 0.239 | 0.0149 | 0.0016 | 6.24E-21 | 0.756 | -0.0101 | 0.023 | 0.3308 |
| rs150537577 | G | A | 0.0805 | 0.0151 | 0.0026 | 3.17E-09 | 0.918 | 0.0488 | 0.0485 | 0.1571 |
| rs62155873 | C | T | 0.8779 | 0.0151 | 0.0021 | 3.23E-13 | 0.112 | 0 | 0.0306 | 0.5 |
| rs12591647 | C | T | 0.1829 | 0.0152 | 0.0018 | 1.53E-17 | 0.83 | -0.0101 | 0.0255 | 0.3468 |
| rs35417702 | C | T | 0.4765 | 0.0152 | 0.0014 | 9.22E-28 | 0.569 | 0 | 0.0153 | 0.5 |
| rs57352738 | T | A | 0.796 | 0.0152 | 0.0017 | 1.93E-19 | 0.203 | 0.0198 | 0.023 | 0.1942 |
| rs9655780 | G | A | 0.1713 | 0.0153 | 0.0019 | 4.05E-16 | 0.823 | 0.01 | 0.0281 | 0.3614 |
| rs55736314 | G | C | 0.401 | 0.0154 | 0.0014 | 1.91E-28 | 0.587 | 0.0101 | 0.0204 | 0.3112 |
| rs6557171 | C | T | 0.6768 | 0.0155 | 0.0015 | 2.49E-25 | 0.295 | 0.0198 | 0.0204 | 0.1659 |
| rs112806496 | G | C | 0.0871 | 0.016 | 0.0025 | 7.77E-11 | 0.928 | 0.0202 | 0.0357 | 0.2858 |
| rs34098770 | G | A | 0.848 | 0.016 | 0.002 | 6.22E-16 | 0.189 | 0.0296 | 0.0281 | 0.1461 |
| rs929511 | C | T | 0.8746 | 0.016 | 0.0021 | 1.28E-14 | 0.136 | -0.0101 | 0.0306 | 0.3713 |
| rs10021733 | C | T | 0.8525 | 0.0164 | 0.0027 | 6.24E-10 | 0.171 | 0.0392 | 0.023 | 0.0438 |
| rs660001 | G | A | 0.7887 | 0.0164 | 0.0017 | 2.53E-22 | 0.233 | -0.0101 | 0.0179 | 0.2868 |
| rs9289300 | C | T | 0.1588 | 0.0164 | 0.0019 | 3.03E-18 | 0.819 | -0.0305 | 0.023 | 0.0923 |
| rs11678980 | G | A | 0.5473 | 0.0166 | 0.0014 | 9.88E-33 | 0.43 | 0 | 0.0204 | 0.5 |
| rs17502934 | G | T | 0.8511 | 0.0167 | 0.002 | 3.41E-17 | 0.148 | 0.0488 | 0.0281 | 0.041 |
| rs7849487 | G | T | 0.3422 | 0.0167 | 0.0015 | 4.32E-29 | 0.637 | 0.0198 | 0.023 | 0.1942 |
| rs72828517 | C | T | 0.1727 | 0.0168 | 0.0019 | 4.70E-19 | 0.856 | 0.01 | 0.0204 | 0.3129 |
| rs61104616 | G | A | 0.4751 | 0.0172 | 0.0014 | 5.40E-35 | 0.531 | 0 | 0.0204 | 0.5 |
| rs13145650 | C | T | 0.083 | 0.0173 | 0.0025 | 2.26E-12 | 0.92 | -0.0305 | 0.0281 | 0.1389 |
| rs72962169 | C | T | 0.8405 | 0.0174 | 0.0019 | 2.65E-20 | 0.168 | 0 | 0.0255 | 0.5 |
| rs72917504 | C | T | 0.9417 | 0.0178 | 0.003 | 1.48E-09 | 0.053 | 0.01 | 0.0434 | 0.4093 |
| rs34720381 | C | T | 0.9093 | 0.018 | 0.0024 | 3.19E-14 | 0.079 | -0.0202 | 0.0357 | 0.2858 |
| rs66568921 | G | T | 0.3559 | 0.0181 | 0.0015 | 7.92E-34 | 0.634 | 0.0198 | 0.0204 | 0.1659 |
| rs1008078 | C | T | 0.5981 | 0.0182 | 0.0014 | 6.12E-39 | 0.425 | -0.0202 | 0.0153 | 0.0934 |
| rs73648455 | C | T | 0.9203 | 0.0183 | 0.0026 | 9.72E-13 | 0.082 | 0.0583 | 0.0383 | 0.0639 |
| rs4352658 | C | T | 0.919 | 0.019 | 0.0026 | 1.36E-13 | 0.089 | 0.01 | 0.0383 | 0.3974 |
| rs79265434 | G | A | 0.1166 | 0.0197 | 0.0022 | 1.71E-19 | 0.892 | 0 | 0.0255 | 0.5 |
| rs56319902 | C | T | 0.7845 | 0.0206 | 0.0017 | 4.26E-34 | 0.24 | -0.0101 | 0.0204 | 0.3112 |
| rs13018640 | C | T | 0.3983 | 0.0215 | 0.0014 | 1.59E-53 | 0.609 | 0.077 | 0.0179 | 0 |
| rs118134876 | C | T | 0.9422 | 0.0223 | 0.003 | 5.29E-14 | 0.048 | 0.0488 | 0.051 | 0.1695 |
| rs115000530 | T | A | 0.0549 | 0.025 | 0.0031 | 3.68E-16 | 0.927 | 0.0296 | 0.0485 | 0.271 |
| rs80257979 | G | T | 0.0323 | 0.0252 | 0.004 | 1.49E-10 | 0.954 | 0.0296 | 0.0561 | 0.2992 |
| rs192436652 | C | T | 0.9732 | 0.0319 | 0.0045 | 6.76E-13 | 0.027 | -0.0202 | 0.0638 | 0.3757 |

SNP: single nucleotide polymorphism; ea: effect allele; oa: other allele, eaf: effect allele frequency, beta: β coefficient; se: standard error; *p*-val: *p* value; exp: exposure; out: outcome

* Rs11212135 was chosen as a proxy variant of rs72486027.

**Table 3**: Detailed information of the " leave-one-out" analysis corresponding to the IVW analysis.

| **SNPs** | **beta** | **se** | ***p*-val** |
| --- | --- | --- | --- |
|  |  |  |  |
| rs10006235 | -0.86618806 | 0.113628834 | 2.48E-14 |
| rs10021733 | -0.864335638 | 0.113851101 | 3.15E-14 |
| rs10046069 | -0.866745186 | 0.113715951 | 2.50E-14 |
| rs10060023 | -0.870548539 | 0.113835282 | 2.05E-14 |
| rs10080647 | -0.85775277 | 0.112805123 | 2.87E-14 |
| rs1008078 | -0.893505259 | 0.113843351 | 4.21E-15 |
| rs10189857 | -0.863902222 | 0.114006429 | 3.52E-14 |
| rs10193498 | -0.864251689 | 0.11347729 | 2.62E-14 |
| rs10215082 | -0.86864661 | 0.113964555 | 2.50E-14 |
| rs10460095 | -0.865215746 | 0.113716652 | 2.77E-14 |
| rs10761251 | -0.873453066 | 0.113884908 | 1.73E-14 |
| rs10772644 | -0.870924219 | 0.113828108 | 1.99E-14 |
| rs10773002 | -0.882943514 | 0.114019246 | 9.65E-15 |
| rs10773208 | -0.876113068 | 0.113788216 | 1.37E-14 |
| rs10798888 | -0.870950521 | 0.11384363 | 2.00E-14 |
| rs10799615 | -0.868982885 | 0.113772402 | 2.21E-14 |
| rs10805383 | -0.862658844 | 0.113480818 | 2.92E-14 |
| rs10830858 | -0.86476557 | 0.113574938 | 2.66E-14 |
| rs10844179 | -0.870469544 | 0.113823927 | 2.05E-14 |
| rs10875121 | -0.889623706 | 0.113336533 | 4.18E-15 |
| rs10892807 | -0.870509739 | 0.113860917 | 2.08E-14 |
| rs10940921 | -0.87051952 | 0.113907572 | 2.13E-14 |
| rs10951590 | -0.872638783 | 0.113840223 | 1.78E-14 |
| rs10979613 | -0.87882883 | 0.113830445 | 1.16E-14 |
| rs10996167 | -0.872789409 | 0.113848491 | 1.77E-14 |
| rs11003463 | -0.871524079 | 0.113778998 | 1.86E-14 |
| rs11023764 | -0.875528426 | 0.113643989 | 1.32E-14 |
| rs11030102 | -0.867410272 | 0.113740241 | 2.42E-14 |
| rs11076962 | -0.869866747 | 0.113872321 | 2.19E-14 |
| rs11081529 | -0.864851855 | 0.113705831 | 2.83E-14 |
| rs11082011 | -0.861560003 | 0.114013894 | 4.14E-14 |
| rs11121177 | -0.863460083 | 0.113675898 | 3.06E-14 |
| rs11130380 | -0.870122641 | 0.113845116 | 2.12E-14 |
| rs11138947 | -0.866279034 | 0.113746717 | 2.62E-14 |
| rs11157931 | -0.861034948 | 0.113786475 | 3.82E-14 |
| rs111852224 | -0.871104228 | 0.11384793 | 1.99E-14 |
| rs11211123 | -0.865558484 | 0.113640289 | 2.60E-14 |
| rs11213482 | -0.870580003 | 0.113770198 | 1.98E-14 |
| rs112603734 | -0.865031256 | 0.113738455 | 2.84E-14 |
| rs112806496 | -0.869992005 | 0.113819083 | 2.11E-14 |
| rs1128956 | -0.872101517 | 0.113810721 | 1.82E-14 |
| rs113520408 | -0.87384486 | 0.113906396 | 1.70E-14 |
| rs113615161 | -0.870697044 | 0.113792936 | 1.99E-14 |
| rs114593137 | -0.866290665 | 0.113714264 | 2.57E-14 |
| rs115000530 | -0.869957301 | 0.113849649 | 2.15E-14 |
| rs11542663 | -0.879186087 | 0.113511408 | 9.53E-15 |
| rs11588857 | -0.85696335 | 0.113712137 | 4.84E-14 |
| rs11598765 | -0.871632892 | 0.113784977 | 1.85E-14 |
| rs11620355 | -0.870125321 | 0.113847778 | 2.12E-14 |
| rs11657979 | -0.867726216 | 0.113569103 | 2.16E-14 |
| rs11663602 | -0.880260289 | 0.113874171 | 1.07E-14 |
| rs1167827 | -0.875647841 | 0.113776953 | 1.40E-14 |
| rs11678980 | -0.875305301 | 0.113986428 | 1.60E-14 |
| rs11724690 | -0.873203243 | 0.113745923 | 1.63E-14 |
| rs117398064 | -0.865879321 | 0.113671341 | 2.59E-14 |
| rs118134876 | -0.868593064 | 0.113785792 | 2.28E-14 |
| rs11871429 | -0.870900785 | 0.113910568 | 2.08E-14 |
| rs11894424 | -0.866236702 | 0.113804796 | 2.71E-14 |
| rs12054166 | -0.868942865 | 0.113786304 | 2.23E-14 |
| rs12076635 | -0.864514072 | 0.114311304 | 3.94E-14 |
| rs12113634 | -0.873638124 | 0.11374379 | 1.58E-14 |
| rs12151248 | -0.877699388 | 0.113387788 | 9.89E-15 |
| rs1220779 | -0.871683316 | 0.113787748 | 1.85E-14 |
| rs12273435 | -0.869681715 | 0.113808175 | 2.14E-14 |
| rs12332731 | -0.870981712 | 0.113888341 | 2.05E-14 |
| rs12375949 | -0.871353441 | 0.114003729 | 2.12E-14 |
| rs12438177 | -0.873888316 | 0.113750196 | 1.56E-14 |
| rs1245829 | -0.872286168 | 0.113820862 | 1.81E-14 |
| rs12468040 | -0.873808516 | 0.113904403 | 1.70E-14 |
| rs12503522 | -0.867207716 | 0.113770333 | 2.49E-14 |
| rs12506221 | -0.866015459 | 0.113839708 | 2.80E-14 |
| rs12519073 | -0.870524258 | 0.113829563 | 2.05E-14 |
| rs12524795 | -0.875117877 | 0.11367069 | 1.37E-14 |
| rs12571549 | -0.86420362 | 0.113789053 | 3.08E-14 |
| rs12574281 | -0.870164817 | 0.113798343 | 2.06E-14 |
| rs12591647 | -0.875033025 | 0.113813054 | 1.49E-14 |
| rs12602286 | -0.864002966 | 0.113619647 | 2.86E-14 |
| rs12614263 | -0.87017336 | 0.11380396 | 2.07E-14 |
| rs12638072 | -0.872565524 | 0.113836201 | 1.79E-14 |
| rs12643771 | -0.868381593 | 0.113898333 | 2.46E-14 |
| rs12646523 | -0.878768207 | 0.113690133 | 1.08E-14 |
| rs12670376 | -0.872059662 | 0.113808422 | 1.82E-14 |
| rs12761761 | -0.878420587 | 0.113733811 | 1.13E-14 |
| rs12765185 | -0.87042174 | 0.113774314 | 2.00E-14 |
| rs12789313 | -0.873837592 | 0.113748877 | 1.56E-14 |
| rs12875339 | -0.876842242 | 0.113656331 | 1.21E-14 |
| rs12888615 | -0.874386811 | 0.113779608 | 1.53E-14 |
| rs12957463 | -0.871225593 | 0.11391463 | 2.04E-14 |
| rs12967010 | -0.87484082 | 0.113691234 | 1.42E-14 |
| rs12981405 | -0.871985851 | 0.113804368 | 1.83E-14 |
| rs1301838 | -0.868494324 | 0.113801334 | 2.32E-14 |
| rs13018640 | -0.838539736 | 0.113360456 | 1.39E-13 |
| rs13050131 | -0.868623343 | 0.11376474 | 2.25E-14 |
| rs13133213 | -0.865242536 | 0.113715515 | 2.77E-14 |
| rs13145650 | -0.878729949 | 0.113651428 | 1.06E-14 |
| rs13163845 | -0.871017257 | 0.113837778 | 1.99E-14 |
| rs13197257 | -0.878739035 | 0.113642281 | 1.05E-14 |
| rs13212041 | -0.871731994 | 0.113790422 | 1.85E-14 |
| rs13240401 | -0.860883229 | 0.113763761 | 3.81E-14 |
| rs13261773 | -0.869477984 | 0.113806396 | 2.17E-14 |
| rs13266287 | -0.86858153 | 0.113774086 | 2.27E-14 |
| rs1329125 | -0.868488418 | 0.113803924 | 2.32E-14 |
| rs13327482 | -0.867063302 | 0.113714966 | 2.44E-14 |
| rs1334297 | -0.887405097 | 0.113981176 | 6.94E-15 |
| rs13422673 | -0.870548638 | 0.11386338 | 2.08E-14 |
| rs137079 | -0.877251209 | 0.113515953 | 1.09E-14 |
| rs1426619 | -0.873403136 | 0.113882169 | 1.73E-14 |
| rs1434630 | -0.874373681 | 0.113791137 | 1.54E-14 |
| rs143743568 | -0.873222791 | 0.113669593 | 1.56E-14 |
| rs150537577 | -0.868776865 | 0.113722533 | 2.18E-14 |
| rs1505676 | -0.876492564 | 0.113603452 | 1.21E-14 |
| rs1542354 | -0.873460449 | 0.113738203 | 1.60E-14 |
| rs1544 | -0.870331545 | 0.11383093 | 2.08E-14 |
| rs1564347 | -0.873638124 | 0.11374379 | 1.58E-14 |
| rs1603460 | -0.868466912 | 0.113783947 | 2.30E-14 |
| rs162445 | -0.876245009 | 0.113655434 | 1.26E-14 |
| rs1689510 | -0.854804659 | 0.113965442 | 6.35E-14 |
| rs17048855 | -0.873836382 | 0.113763313 | 1.58E-14 |
| rs17110109 | -0.870364036 | 0.113804561 | 2.04E-14 |
| rs17144467 | -0.870250755 | 0.113867631 | 2.13E-14 |
| rs17148998 | -0.871991251 | 0.113804665 | 1.83E-14 |
| rs1717204 | -0.869472962 | 0.113798417 | 2.16E-14 |
| rs17248751 | -0.867631285 | 0.113814172 | 2.47E-14 |
| rs173003 | -0.86153917 | 0.113439121 | 3.08E-14 |
| rs1738050 | -0.864717356 | 0.113578286 | 2.67E-14 |
| rs17411339 | -0.87121119 | 0.113945433 | 2.08E-14 |
| rs17428076 | -0.862869084 | 0.113645595 | 3.13E-14 |
| rs1747714 | -0.872529408 | 0.113834218 | 1.79E-14 |
| rs1747817 | -0.865051688 | 0.113598601 | 2.64E-14 |
| rs17489649 | -0.875817791 | 0.113806096 | 1.41E-14 |
| rs17502934 | -0.864745456 | 0.113749484 | 2.91E-14 |
| rs175325 | -0.872319892 | 0.113822714 | 1.80E-14 |
| rs17551064 | -0.871018844 | 0.113869584 | 2.02E-14 |
| rs17563464 | -0.872562226 | 0.11383602 | 1.79E-14 |
| rs17565975 | -0.872529408 | 0.113834218 | 1.79E-14 |
| rs17598675 | -0.870672381 | 0.113924037 | 2.13E-14 |
| rs17604349 | -0.873945889 | 0.11377864 | 1.58E-14 |
| rs176218 | -0.860183118 | 0.113869324 | 4.22E-14 |
| rs17732878 | -0.872154689 | 0.113813642 | 1.82E-14 |
| rs178183 | -0.873441932 | 0.113884297 | 1.73E-14 |
| rs17882802 | -0.870182061 | 0.113805917 | 2.07E-14 |
| rs1842713 | -0.876087127 | 0.113680895 | 1.29E-14 |
| rs1861786 | -0.869817405 | 0.113855145 | 2.18E-14 |
| rs1865955 | -0.87784638 | 0.113680419 | 1.14E-14 |
| rs1866823 | -0.868364701 | 0.113828881 | 2.37E-14 |
| rs1890132 | -0.870247932 | 0.113816083 | 2.07E-14 |
| rs192436652 | -0.873673327 | 0.113790498 | 1.62E-14 |
| rs1933264 | -0.868864468 | 0.113792929 | 2.25E-14 |
| rs1955250 | -0.869285796 | 0.11377153 | 2.16E-14 |
| rs1991585 | -0.880694832 | 0.113411808 | 8.13E-15 |
| rs2007655 | -0.87020048 | 0.113809886 | 2.07E-14 |
| rs2011603 | -0.871734427 | 0.113790556 | 1.85E-14 |
| rs2034631 | -0.868475956 | 0.113781473 | 2.30E-14 |
| rs2052285 | -0.870577016 | 0.113839186 | 2.05E-14 |
| rs2055940 | -0.878262532 | 0.113420791 | 9.68E-15 |
| rs2081652 | -0.870785957 | 0.113896843 | 2.08E-14 |
| rs2131167 | -0.8685725 | 0.113760047 | 2.26E-14 |
| rs2179152 | -0.884986591 | 0.113655154 | 6.88E-15 |
| rs2183271 | -0.875051032 | 0.113638223 | 1.36E-14 |
| rs2199409 | -0.870489879 | 0.113804527 | 2.03E-14 |
| rs2212430 | -0.873639992 | 0.113895161 | 1.71E-14 |
| rs2216144 | -0.862594888 | 0.11355384 | 3.05E-14 |
| rs2256965 | -0.78325838 | 0.096529125 | 4.89E-16 |
| rs2287838 | -0.87044745 | 0.113849022 | 2.08E-14 |
| rs2290601 | -0.877308709 | 0.113852698 | 1.30E-14 |
| rs2297293 | -0.878144255 | 0.113485543 | 1.01E-14 |
| rs232496 | -0.873737172 | 0.113746297 | 1.57E-14 |
| rs2364544 | -0.878453522 | 0.113629066 | 1.07E-14 |
| rs2434672 | -0.873787211 | 0.113747578 | 1.57E-14 |
| rs2436760 | -0.870069344 | 0.113798697 | 2.08E-14 |
| rs2447097 | -0.862717181 | 0.113544874 | 3.01E-14 |
| rs2469226 | -0.875150737 | 0.113582873 | 1.31E-14 |
| rs2496482 | -0.870831483 | 0.114017747 | 2.21E-14 |
| rs252991 | -0.872154689 | 0.113813642 | 1.82E-14 |
| rs2545795 | -0.866590922 | 0.114001509 | 2.93E-14 |
| rs2554835 | -0.870220253 | 0.11381393 | 2.07E-14 |
| rs2570497 | -0.868447836 | 0.113871716 | 2.41E-14 |
| rs2725370 | -0.876756025 | 0.11383435 | 1.34E-14 |
| rs2764684 | -0.865580865 | 0.11375545 | 2.76E-14 |
| rs2838006 | -0.870998787 | 0.113921888 | 2.08E-14 |
| rs28513882 | -0.870548638 | 0.11386338 | 2.08E-14 |
| rs2885198 | -0.870220253 | 0.11381393 | 2.07E-14 |
| rs2923424 | -0.879108173 | 0.113841722 | 1.14E-14 |
| rs2929032 | -0.874142657 | 0.113736333 | 1.52E-14 |
| rs2958182 | -0.866915805 | 0.113678704 | 2.42E-14 |
| rs2964199 | -0.867447743 | 0.113735433 | 2.40E-14 |
| rs2964255 | -0.868485337 | 0.113779017 | 2.29E-14 |
| rs2989476 | -0.867122611 | 0.11377981 | 2.52E-14 |
| rs2998299 | -0.870948216 | 0.11385998 | 2.02E-14 |
| rs3026996 | -0.86842939 | 0.113924833 | 2.48E-14 |
| rs303752 | -0.864007798 | 0.113555036 | 2.77E-14 |
| rs3111251 | -0.863470289 | 0.11343222 | 2.69E-14 |
| rs34067381 | -0.872458202 | 0.113830309 | 1.79E-14 |
| rs34098770 | -0.86805715 | 0.113849548 | 2.45E-14 |
| rs34305371 | -0.874809553 | 0.113959273 | 1.63E-14 |
| rs34394051 | -0.869472962 | 0.113798417 | 2.16E-14 |
| rs34720381 | -0.874725265 | 0.113749697 | 1.47E-14 |
| rs34807077 | -0.873402286 | 0.113771205 | 1.63E-14 |
| rs35417702 | -0.87761797 | 0.114112944 | 1.46E-14 |
| rs35606437 | -0.87048204 | 0.113825782 | 2.05E-14 |
| rs35929923 | -0.874124209 | 0.11377047 | 1.55E-14 |
| rs363096 | -0.868581646 | 0.113943409 | 2.48E-14 |
| rs3751331 | -0.87518288 | 0.11376243 | 1.44E-14 |
| rs3809634 | -0.872638783 | 0.113840223 | 1.78E-14 |
| rs382196 | -0.867059154 | 0.113688195 | 2.41E-14 |
| rs3859523 | -0.873826953 | 0.113774929 | 1.59E-14 |
| rs3897821 | -0.855763258 | 0.113637354 | 5.05E-14 |
| rs3948495 | -0.871967704 | 0.113803371 | 1.83E-14 |
| rs401526 | -0.868456509 | 0.113822577 | 2.35E-14 |
| rs401966 | -0.865207604 | 0.113719452 | 2.78E-14 |
| rs42210 | -0.876355881 | 0.113549222 | 1.18E-14 |
| rs42302 | -0.868505108 | 0.113774163 | 2.28E-14 |
| rs4298514 | -0.868375661 | 0.113820557 | 2.36E-14 |
| rs4320563 | -0.86482243 | 0.113768613 | 2.93E-14 |
| rs4328757 | -0.868419719 | 0.113799187 | 2.33E-14 |
| rs4352658 | -0.871320595 | 0.113840303 | 1.95E-14 |
| rs4358081 | -0.879687453 | 0.113272806 | 8.09E-15 |
| rs4369924 | -0.874568596 | 0.113706227 | 1.45E-14 |
| rs4384309 | -0.872388363 | 0.113826474 | 1.80E-14 |
| rs4467547 | -0.858091903 | 0.113438741 | 3.90E-14 |
| rs4500930 | -0.866377103 | 0.113768087 | 2.63E-14 |
| rs4663617 | -0.862198426 | 0.113315179 | 2.77E-14 |
| rs4673840 | -0.872332239 | 0.113823392 | 1.80E-14 |
| rs4675248 | -0.870305961 | 0.113826594 | 2.08E-14 |
| rs4719944 | -0.870808182 | 0.113941097 | 2.13E-14 |
| rs4726070 | -0.870764015 | 0.113894154 | 2.08E-14 |
| rs4757957 | -0.868500508 | 0.113909686 | 2.45E-14 |
| rs4766424 | -0.874620526 | 0.113720067 | 1.46E-14 |
| rs4778058 | -0.874845437 | 0.113753607 | 1.46E-14 |
| rs4787457 | -0.855572248 | 0.113951731 | 5.99E-14 |
| rs4793090 | -0.870045161 | 0.11382926 | 2.12E-14 |
| rs4810894 | -0.865376663 | 0.113576127 | 2.55E-14 |
| rs4839155 | -0.861472412 | 0.113354279 | 2.97E-14 |
| rs4846724 | -0.872751238 | 0.113846396 | 1.77E-14 |
| rs4848924 | -0.872458202 | 0.113830309 | 1.79E-14 |
| rs488476 | -0.862560589 | 0.1138059 | 3.48E-14 |
| rs4895650 | -0.8684418 | 0.113791483 | 2.31E-14 |
| rs4899012 | -0.864253496 | 0.113612803 | 2.81E-14 |
| rs4904523 | -0.870180918 | 0.113802154 | 2.07E-14 |
| rs4915735 | -0.875564507 | 0.113546649 | 1.25E-14 |
| rs4977885 | -0.871821255 | 0.113795326 | 1.84E-14 |
| rs4984541 | -0.867867758 | 0.113744805 | 2.35E-14 |
| rs55736314 | -0.871635621 | 0.113989553 | 2.06E-14 |
| rs56099375 | -0.870687727 | 0.113831491 | 2.03E-14 |
| rs56319902 | -0.881899435 | 0.114018229 | 1.04E-14 |
| rs56391344 | -0.873559951 | 0.113890771 | 1.72E-14 |
| rs56794817 | -0.868142583 | 0.113768718 | 2.33E-14 |
| rs57349798 | -0.872090997 | 0.113810143 | 1.82E-14 |
| rs57352738 | -0.869093294 | 0.113923702 | 2.37E-14 |
| rs575113 | -0.873624901 | 0.113894333 | 1.71E-14 |
| rs5754753 | -0.870878339 | 0.113904976 | 2.08E-14 |
| rs57661533 | -0.868697456 | 0.113754063 | 2.23E-14 |
| rs580652 | -0.874196288 | 0.113725069 | 1.51E-14 |
| rs590013 | -0.866402404 | 0.113731088 | 2.58E-14 |
| rs6043521 | -0.870189476 | 0.113804088 | 2.07E-14 |
| rs6065080 | -0.868482772 | 0.113899938 | 2.44E-14 |
| rs6065784 | -0.868442309 | 0.113839299 | 2.37E-14 |
| rs61104616 | -0.875651481 | 0.114005383 | 1.58E-14 |
| rs6123924 | -0.870784954 | 0.113826766 | 2.01E-14 |
| rs61527214 | -0.881815281 | 0.113612331 | 8.39E-15 |
| rs61997667 | -0.866679327 | 0.113764043 | 2.57E-14 |
| rs62155350 | -0.873969544 | 0.113620863 | 1.45E-14 |
| rs62155873 | -0.872342564 | 0.113823959 | 1.80E-14 |
| rs62174974 | -0.870522949 | 0.113807545 | 2.02E-14 |
| rs62179650 | -0.874917391 | 0.113778779 | 1.48E-14 |
| rs62182994 | -0.873139174 | 0.113867686 | 1.75E-14 |
| rs62379838 | -0.878077581 | 0.11356433 | 1.06E-14 |
| rs628993 | -0.872596256 | 0.113644396 | 1.61E-14 |
| rs6449503 | -0.873490796 | 0.114214723 | 2.04E-14 |
| rs6490618 | -0.863172333 | 0.113375984 | 2.67E-14 |
| rs6493265 | -0.87078774 | 0.113894125 | 2.08E-14 |
| rs6557171 | -0.868723803 | 0.113986385 | 2.51E-14 |
| rs660001 | -0.880802426 | 0.113939489 | 1.07E-14 |
| rs66568921 | -0.869200037 | 0.114088922 | 2.56E-14 |
| rs66721975 | -0.882361345 | 0.112697135 | 4.90E-15 |
| rs6690195 | -0.872789409 | 0.113848491 | 1.77E-14 |
| rs6697584 | -0.874696059 | 0.113787786 | 1.51E-14 |
| rs6704768 | -0.861903239 | 0.113532578 | 3.16E-14 |
| rs6731373 | -0.868900049 | 0.113827696 | 2.29E-14 |
| rs6812533 | -0.872129758 | 0.113812272 | 1.82E-14 |
| rs68145588 | -0.872941783 | 0.113763283 | 1.68E-14 |
| rs6917154 | -0.87184029 | 0.113796372 | 1.84E-14 |
| rs6924023 | -0.867669695 | 0.11385927 | 2.53E-14 |
| rs6977237 | -0.863621626 | 0.113484849 | 2.74E-14 |
| rs6994287 | -0.878246235 | 0.113503604 | 1.01E-14 |
| rs7012546 | -0.866617198 | 0.113707056 | 2.51E-14 |
| rs7029718 | -0.85177499 | 0.114319398 | 9.28E-14 |
| rs7041702 | -0.870579682 | 0.113837239 | 2.05E-14 |
| rs7167688 | -0.870305961 | 0.113826594 | 2.08E-14 |
| rs7171405 | -0.868922269 | 0.113796678 | 2.25E-14 |
| rs717996 | -0.88336935 | 0.11343422 | 6.83E-15 |
| rs7226824 | -0.87015007 | 0.113794606 | 2.06E-14 |
| rs11212135 | -0.870485505 | 0.113823961 | 2.05E-14 |
| rs7254263 | -0.87302563 | 0.113861456 | 1.75E-14 |
| rs72622559 | -0.870720083 | 0.113855377 | 2.05E-14 |
| rs72672052 | -0.868022505 | 0.113738266 | 2.32E-14 |
| rs72709560 | -0.871849864 | 0.113796898 | 1.84E-14 |
| rs72828517 | -0.872160137 | 0.114037178 | 2.04E-14 |
| rs72829857 | -0.875901899 | 0.113826907 | 1.41E-14 |
| rs72917504 | -0.871053009 | 0.113805636 | 1.95E-14 |
| rs72944064 | -0.87609889 | 0.113645392 | 1.27E-14 |
| rs72962169 | -0.873911857 | 0.11391007 | 1.69E-14 |
| rs72993796 | -0.865677172 | 0.113503184 | 2.40E-14 |
| rs730384 | -0.865072238 | 0.113735445 | 2.83E-14 |
| rs73055556 | -0.876066835 | 0.113324157 | 1.07E-14 |
| rs73191311 | -0.870207012 | 0.113856287 | 2.12E-14 |
| rs7321274 | -0.873444776 | 0.113763334 | 1.62E-14 |
| rs7326331 | -0.86604353 | 0.113832528 | 2.78E-14 |
| rs73344830 | -0.865437889 | 0.113992931 | 3.15E-14 |
| rs736281 | -0.873540446 | 0.113741358 | 1.59E-14 |
| rs73648455 | -0.86634751 | 0.113717408 | 2.57E-14 |
| rs743316 | -0.871985851 | 0.113804368 | 1.83E-14 |
| rs74415461 | -0.869483053 | 0.113793791 | 2.16E-14 |
| rs7449561 | -0.871753983 | 0.11379163 | 1.85E-14 |
| rs7460106 | -0.865580597 | 0.11357229 | 2.51E-14 |
| rs746839 | -0.863413046 | 0.113756838 | 3.20E-14 |
| rs74787922 | -0.872221938 | 0.113817335 | 1.81E-14 |
| rs7575637 | -0.873160223 | 0.113868841 | 1.75E-14 |
| rs7603132 | -0.869218331 | 0.113852335 | 2.27E-14 |
| rs76076331 | -0.874859702 | 0.113839371 | 1.53E-14 |
| rs7617204 | -0.866818516 | 0.113861684 | 2.68E-14 |
| rs76235882 | -0.874578429 | 0.113671633 | 1.43E-14 |
| rs7650602 | -0.882382394 | 0.113284994 | 6.75E-15 |
| rs76577427 | -0.876291526 | 0.113776752 | 1.34E-14 |
| rs7672622 | -0.870319529 | 0.113796334 | 2.04E-14 |
| rs7683416 | -0.871354168 | 0.114007764 | 2.12E-14 |
| rs76878669 | -0.864223595 | 0.113673351 | 2.90E-14 |
| rs7692359 | -0.870551434 | 0.113833371 | 2.05E-14 |
| rs77025239 | -0.873828031 | 0.113783936 | 1.59E-14 |
| rs77609760 | -0.868602783 | 0.113993577 | 2.54E-14 |
| rs7849487 | -0.869276115 | 0.113968449 | 2.40E-14 |
| rs78648104 | -0.874448356 | 0.113664044 | 1.43E-14 |
| rs7875078 | -0.865717951 | 0.113554567 | 2.46E-14 |
| rs7894722 | -0.870191101 | 0.113807892 | 2.07E-14 |
| rs790647 | -0.873441932 | 0.113884297 | 1.73E-14 |
| rs7910403 | -0.870715129 | 0.113834891 | 2.03E-14 |
| rs7924036 | -0.885104241 | 0.113671373 | 6.89E-15 |
| rs79265434 | -0.874841726 | 0.113961036 | 1.63E-14 |
| rs7972246 | -0.872675925 | 0.113842262 | 1.78E-14 |
| rs79728014 | -0.870826948 | 0.113817555 | 1.99E-14 |
| rs7974852 | -0.875208877 | 0.113787418 | 1.45E-14 |
| rs7977614 | -0.865963344 | 0.113854295 | 2.83E-14 |
| rs79994730 | -0.86487185 | 0.113677477 | 2.78E-14 |
| rs8008382 | -0.871844115 | 0.113796582 | 1.84E-14 |
| rs8024 | -0.870639398 | 0.113845152 | 2.05E-14 |
| rs80257979 | -0.870135394 | 0.113820695 | 2.09E-14 |
| rs8030487 | -0.871849864 | 0.113796898 | 1.84E-14 |
| rs806816 | -0.873533338 | 0.113754786 | 1.60E-14 |
| rs8097125 | -0.87020048 | 0.113809886 | 2.07E-14 |
| rs912883 | -0.870210197 | 0.113811898 | 2.07E-14 |
| rs9289300 | -0.881915503 | 0.113598599 | 8.27E-15 |
| rs929511 | -0.87394229 | 0.113796486 | 1.59E-14 |
| rs9359939 | -0.868910589 | 0.113805244 | 2.26E-14 |
| rs936496 | -0.878110428 | 0.113399604 | 9.67E-15 |
| rs9373363 | -0.870579682 | 0.113837239 | 2.05E-14 |
| rs9375188 | -0.869680581 | 0.114381665 | 2.89E-14 |
| rs9411331 | -0.865593541 | 0.113888734 | 2.95E-14 |
| rs9492774 | -0.871710224 | 0.113789226 | 1.85E-14 |
| rs9529146 | -0.86684404 | 0.113801919 | 2.59E-14 |
| rs9556958 | -0.878950026 | 0.113636731 | 1.04E-14 |
| rs9616947 | -0.869272497 | 0.113846916 | 2.25E-14 |
| rs9633970 | -0.873123423 | 0.113743854 | 1.64E-14 |
| rs9655780 | -0.871158589 | 0.113865143 | 2.00E-14 |
| rs969512 | -0.876690799 | 0.113653995 | 1.22E-14 |
| rs9853928 | -0.868922621 | 0.113856599 | 2.32E-14 |
| rs9859556 | -0.904435784 | 0.114329794 | 2.56E-15 |
| rs9882532 | -0.864429283 | 0.113835067 | 3.11E-14 |
| rs9886703 | -0.870948216 | 0.11385998 | 2.02E-14 |
| rs9927137 | -0.858118784 | 0.113059817 | 3.20E-14 |
| rs9927842 | -0.873225677 | 0.113715967 | 1.60E-14 |
| rs9929556 | -0.870372465 | 0.113837574 | 2.08E-14 |
| rs9929762 | -0.870312897 | 0.113830854 | 2.08E-14 |
| rs9933256 | -0.879751585 | 0.113659398 | 9.92E-15 |
| All | -0.870628471 | 0.113576796 | 1.78E-14 |

The results of the leave-one-out analysis demonstrated no potentially influential SNPs driving the causal link between educational attainment and RA in the replication practice ( *p*-val < 0.05 ). SNP: single nucleotide polymorphism; beta: β coefficient; se: standard error;

**Table 4:** The SNPs and their corresponding phenotypes considered as confounders

| **SNPs** | **Confounders** |
| --- | --- |
| rs2011603 | Type 2 diabetes |
| rs2725370 | Type 2 diabetes |
| rs7029718 | Type 2 diabetes |
| rs9927842 | Type 2 diabetes |
| rs1008078 | Smoking initiation |
| rs11076962 | Smoking initiation |
| rs11678980 | Smoking initiation |
| rs3897821 | Smoking initiation |
| rs4500930 | Smoking initiation |
| rs56099375 | Smoking initiation |
| rs62155873 | Smoking initiation |
| rs10805383 | Body Mass Index (BMI) |
| rs11030102 | Body Mass Index (BMI) |
| rs1167827 | Body Mass Index (BMI) |
| rs13163845 | Body Mass Index (BMI) |
| rs34720381 | Body Mass Index (BMI) |
| rs78648104 | Body Mass Index (BMI) |
| rs7924036 | Body Mass Index (BMI) |
